# A Metabolome Wide Association Study of Fruit and Vegetable Consumption and Associations with Cardiovascular Disease Risk Factors: The International Study of Macro-/Micronutrients and Blood Pressure (INTERMAP) Study

**DOI:** 10.1016/j.tjnut.2024.11.004

**Published:** 2024-11-12

**Authors:** Linda M Oude Griep, Elena Chekmeneva, Linda Van Horn, Queenie Chan, Martha L Daviglus, Gary Frost, Elaine Holmes, Timothy MD Ebbels, Paul Elliott

**Affiliations:** 1 Medical Research Council (MRC) Epidemiology Unit, University of Cambridge, Cambridge, United Kingdom; 2 Department of Epidemiology and Biostatistics, School of Public Health, Imperial College London, London, United Kingdom; 3 Department of Metabolism, Digestion and Reproduction, Imperial College London, London, United Kingdom; 4 Department of Preventive Medicine, Feinberg School of Medicine, Northwestern University, Chicago, Illinois, United States; 5 Institute for Minority Health Research, University of Illinois, Chicago, Illinois, United States; 6 Institute of Health Futures, Murdoch University, Perth, Western Australia, Australia; 7 MRC Centre for Environment and Health, Imperial College London, London, United Kingdom

**Keywords:** Biomarkers, blood pressure, body mass index, cardiovascular disease risk factors, 24-h dietary recalls, fruit and vegetables, urinary metabolites

## Abstract

**Background:**

Epidemiologic evidence linking blood pressure (BP) and body weight-lowering effects with fruit and vegetable consumption mostly relies on self-reported dietary assessment prone to misreport and under- or overestimation of relationships.

**Objectives:**

We aimed to characterize objective 24-h urinary metabolites and a derived metabolite score associated with fruit and vegetable intake and assessed their associations with BP and BMI, with validation across cohorts.

**Methods:**

We used untargeted proton nuclear magnetic resonance spectroscopy (^1^H NMR) of 2 timed repeated 24-h urine collections from free-living participants from the US (*n* = 2032) and the UK (*n* = 449) of the cross-sectional International Study of Macro-/Micronutrients and Blood Pressure (INTERMAP). We evaluated correlations between fruit and vegetable intake assessed by 24-h dietary recalls with 7100 ^1^H NMR features, adjusted for confounders and multiple testing. We related identified metabolites and a metabolite score with BP and BMI using extensively adjusted multiple linear regression models.

**Results:**

We characterized 11 ^1^H NMR-derived 24-h urinary metabolites related to fruit and vegetable intake, reproducible across multiple 24-h urine collections of both cohorts. Proline betaine, citrate, N-methylproline, scyllo-inositol, 2-hydroxy-2-(4-methyl cyclohex-3-en-1-yl) propoxyglucuronide, and proline were associated with fruit intake, specifically with *Rutaceae* intake, whereas S-methyl-L-cysteine sulfoxide and S-methyl-L-cysteine sulfoxide metabolite were associated with *Brassicaceae* intake. The metabolite score, explaining 39.8% of fruit and vegetable intake, was inversely associated with systolic BP [−1.65 mmHg; 95% confidence interval (CI): −2.68, −0.62; *P* < 0.002] and BMI (−1.21 kg/m^2^; 95% CI: −1.62, −0.78; *P* < 0.0001). These associations were, to a large extent, explained by urinary citrate excretion.

**Conclusions:**

We identified ^1^H NMR-derived urinary metabolites associated with fruit and vegetable consumption, consistent and reproducible between urine collections and across populations. A higher fruit and vegetable-related metabolite score showed associations with lower systolic BP and BMI, mainly mediated by citrate, but would need confirmation in further studies.

## Introduction

Despite significant advances, cardiovascular diseases continue to be the leading cause of disability and premature death worldwide [1]. High blood pressure (BP), suboptimal diet quality—particularly low fruit and vegetable consumption—and high BMI are the leading modifiable risk factors [1]. The benefits of higher fruit and vegetable intakes on high BP and weight loss are well-established [2,3]. Traditionally, however, this evidence predominantly relied on self-reported dietary assessments, which are vulnerable to errors such as underreporting, recall inaccuracies, and misclassification of portion sizes [4]. These limitations can weaken statistical power and introduce biases in various directions, affecting the reliability of diet-disease relationships [5]. This highlights the emerging need for objective measurement of fruit and vegetable intake and associations with BP and BMI.

Acknowledging the limitations of self-reported dietary assessment tools and recent advances in high-throughput technologies, there has been accelerated progress in identifying objective biomarkers supplementing established biomarkers such as carotenoids and urinary potassium to assess fruit and vegetable intake [6]. Utilizing metabolomics, which measures and characterizes a wide range of small, low-molecular weight metabolites in biofluids, allows simultaneous reflection of individuals’ dietary intakes and variability in metabolism and may objectively measure adherence and metabolic responses to dietary intake [7]. In controlled feeding studies, metabolic profiling has facilitated the categorization of individuals by (un)healthy dietary patterns [8], whereas specific metabolites have been identified as objective markers of specific food intakes, e.g., proline betaine for citrus fruits [9] and S-methyl-L-cyteine sulfoxide (SMCSO) for cruciferous vegetables [10]. Although targeted metabolomics has been employed to identify populations’ adherence to various dietary patterns [11,12], the application of untargeted metabolomics to identify biomarkers of fruit and vegetable intake—and its botanical families with significant differences in biochemical compounds—and to assess objective associations with BP and BMI in large epidemiologic studies, involving free-living populations is yet unexplored. The findings have potential to provide insights into underlying biological pathways, which will advance our understanding of how fruit and vegetable types may influence cardiometabolic risk.

Hence, we characterized metabolic signatures of fruit and vegetable consumption using proton nuclear magnetic resonance (^1^H NMR) spectroscopy of 2-timed repeated 24-h urine collections of 2481 free-living individuals from the population-based International Study of Macro-and Micronutrients and Blood Pressure (INTERMAP) study. We then used the identified metabolites and available urinary potassium to build a fruit and vegetable-related metabolite score. Subsequently, we examined cross-sectional associations between identified fruit and vegetable-related metabolites and the metabolite score with BP and BMI.

## Methods

### Study population

The cross-sectional INTERMAP study surveyed 4680 men and women aged 40–59 y from Japan, the People’s Republic of China, the United Kingdom, and the United States to investigate dietary and other factors associated with BP [13]. All 4680 participants attended 4 clinic visits: 2 visits on consecutive days and 2 visits on consecutive days approximately 3 wk later (Supplemental Figure 1). Data obtained included 4 in-depth 24-h multipass dietary recalls, 2-timed 24-h urine collections, 8 BP measurements, and anthropometric and extensive questionnaire information on lifestyle, medical history, and medication use.

This analyses relates to data from Western population samples with generally homogenous urinary metabolic phenotypes [14] and complete dietary as well as ^1^H NMR data; 2032 US individuals from 8 population samples for discovery analyses and 449 UK individuals from 2 population samples to replicate findings. The flowchart of participants by exclusion criteria in this study is described in the Supplemental methods and presented in Supplemental Figure 2. The INTERMAP study received institutional review board approval from Northwestern University (STU00204462-CR0002) and Research Ethics Committee approval of the Health Research Authority (United Kingdom, #EC3169); all participants gave written informed consent. INTERMAP is registered at www.clinicaltrials.gov as NCT00005271.

### Dietary assessment

Dietary data of all participants were collected with 4 in-depth 24-h multipass dietary recalls by trained interviewers using standardized procedures [15]. Dietary data were computerized (Nutrition Data System, version 2.91; University of Minnesota). Nutrient intakes (excluding dietary supplement intakes) were computed by multiplying the amount consumed by its nutrient content using country-specific food tables standardized across countries by the Nutrition Coordinating Center, University of Minnesota [15]. Reported fruit and vegetable items were classified according to Stern’s botanical classification of fruits and seeds [16] comprising fruits (including 100% juices) and vegetables (excluding potatoes). Individual fruit and vegetable items were further categorized into total fruit and vegetables, by subgroup (fruit and vegetables) and the 5 mostly consumed botanical families (Supplemental Table 1: Rutaceae, Rosaceae, Solanaceae, Cucurbiceae, and Brassicaceae).

### BP and anthropometric measurements

Systolic and diastolic BP (first and fifth Korotkoff sounds, respectively) were measured by trained staff with a random-zero sphygmomanometer with availability of 3 cuff sizes. BP was measured twice at each study visit, 8 times in total. Measurements were carried out on the right arm with the participant seated after a rest of ≥5 min in a quiet room with bladder empty, feet flat on the floor, arm at heart level, and no physical activity, eating, drinking, or smoking in the preceding half hour. During 2 visits, body weight and height without shoes and heavy clothing were measured, and average BMI (kg/m^2^) was calculated.

### Urine collection

Each participant provided 2 borate-preserved timed 24-h urine collections at the second and fourth study visits, respectively. Aliquots were frozen on site (−20^o^C) and air-freighted frozen to the Central Laboratory, Leuven, Belgium, for electrolyte analyses with 8% of aliquots sent blinded to estimate technical error [13]; urinary potassium and sodium were measured using emission flame photometry. In the total INTERMAP population, Pearson partial correlation coefficients, adjusted for sample and sex, between reported intakes by 24-h recall and 24-h urinary excretions were 0.42 for sodium and 0.55 for potassium [15].

### Proton nuclear magnetic resonance metabolic profiling of urine

Untargeted metabolomic profiling was performed using high-resolution ^1^H NMR spectroscopy using a Bruker Avance III spectrometer (Bruker Biospin), operating at 600 MHz, equipped with a 5 mm, TCI, Z-gradient CryoProbe following a previously published protocol [17]. Free induction decays were Fourier-transformed using 3-trimethylsilyl propionic acid as internal standard and were baseline- and phase-corrected by use of inhouse software. The spectral regions containing residual water and urea (δ 4.5–6.4), TSP (δ −0.2 to 0.2, δ 0.2–0.5, −4.5 to −0.2, and δ 9.5–15.5) were removed. The remaining spectral data points were reduced to 7100 variables (features) corresponding to the regions δ 0.5–9.5 using bin widths of 0.001 ppm and normalized using probabilistic quotient normalization [18]. Metabolic outliers were identified and excluded from further analyses by use of Hotelling’s T^2^ statistic on the scores of principal component analysis (Supplemental Methods, Supplemental Figure 2). Quality control showed high analytical reproducibility (>98%) after blind analyses of split randomized urine samples [19].

### Identification of urinary metabolites related to fruit and vegetable intake

Combinations of statistical and analytical approaches were used to identify potential chemical structure of urinary ^1^H NMR metabolites significantly related to fruit and vegetable intake. Structural correlations between significant individual ^1^H NMR spectral variables were identified using statistical total correlation spectroscopy [20] and subset optimization by reference matching [21] to provide information on the potential molecular structure. Together with an expert in structural elucidation of metabolites and in NMR spectroscopy (EC), we performed a range of 2-dimensional nuclear magnetic resonance experiments on a Bruker 800 MHz spectrometer including ^1^H-^1^H total correlation, ^1^H-^1^H correlation, 2D *J*-resolved, and 2D ^1^H-^13^C heteronuclear single quantum coherence spectroscopy on a pooled 24-h urine sample of 5 participants with high signal intensities for spectral features of interest. We used Matlab R2021b (Mathworks Inc.) and TopSpin 3.1 (Bruker BioSpin) for spectral investigations. Obtained structural information was compared with available inhouse and publicly available databases, e.g., Human Metabolome Database [22], as well as previously published data on urinary ^1^H NMR metabolites, including proline betaine [9,23], SMCSO and its metabolite [10,24,25], and 2-hydroxy-2-(4-methyl cyclohex-3-en-1-yl) propoxyglucuronide) [26,27]. We performed spike-in experiments when the chemical standards were available to confirm metabolite identities. The criteria of the Metabolomics Standards Initiative [28] were used to report strategies and level of confidence in assignment of identified metabolites (Supplemental Table 3).

### Statistical analyses

Energy-adjusted dietary recall data (g/1000 kcal) were averaged across the first, second, third, and fourth 24-h dietary recalls to capture urinary excretions within 24–48 h. Baseline characteristics were calculated for individual and pooled cohorts stratified by higher and lower fruit and vegetable intake using the median, offering a study-specific measure reflecting the distribution of intake within this study population. From the means of the first and second pairs of visits, we estimated the reliability of total fruit and vegetable intake for individuals using the following formula: 1/[1+ratio/2)]×100, in which the ratio is within-participant divided by between participant variance [16,17]. This gives an indication of the effect of day-to-day variability on the associations with health outcomes [29,30].

We applied orthogonal partial least-squares discriminant analysis to explore patterns of the 7100 ^1^H NMR spectral features that discriminated the highest (Q4) from the lowest (Q1) country-specific quartiles of fruit and vegetable consumers. To include the total population and to allow for adjustment for potential confounding factors, we then calculated standardized partial correlations between averaged fruit and vegetable intake (g/1000 kcal) from the first and second 2 24-h dietary recalls with all 7100 ^1^H NMR spectral features of the corresponding 24-h urinary collection, using the US data for discovery and UK data for replication. To correct for multiple testing, we applied the Metabolome Wide Significance Level [31] (*P*≤4×10^-6^) for discovery analysis and a false discovery rate [32] threshold of 5% for replication in the UK population with smaller sample size and thus limited power. Only the significant correlations were investigated if adjacent spectral variables passed the country-specific significance threshold and if correlation coefficients of adjacent spectral variables were in the same direction. Associations were considered reproducible if significance of associations was prevalent in both visits and across populations.

Country-specific intraclass correlation coefficients (ICC) with 95% CIs were calculated using linear mixed models with random intercept to assess the reproducibility between the 2 24-h urinary metabolite excretions collected approximately 3 wk apart [33].

We used stepwise regression to identify a model of urinary metabolites and potassium with best predictive value of fruit and vegetable intake. From the stepwise regression findings, we derived metabolite score (*z*-score) for fruit and vegetable intake. First, each metabolite was standardized by subtracting the mean of the total population from the individual mean and divided by the SD. Second, standardized values for urinary metabolites were summed. Third, the sum of standardized values was divided by the total number of individual metabolites included in the score.

In addition, we performed partial correlation analysis between the averaged fruit and vegetable intake (total, by group and botanical family) from 4 24-h dietary recalls with averaged individual metabolites, potassium, and metabolite scores from 2 24-h urinary collections, adjusted for age, gender, and population sample, for the United States and United Kingdom separately and pooled cohorts.

We then performed multiple regression analyses using averaged data to examine associations of individual metabolites, potassium, and the metabolite score with systolic and diastolic BP and BMI. We used a 2SD increase, allowing standardized comparison between effect sizes of the exposures, capturing potentially clinically meaningful differences while aligning with established research practices for comparability. Models were fitted by country, and coefficients were pooled, weighted by the inverse of their variance [30,34]. Cross-country heterogeneity of regression coefficients was assessed by chi-square test. For the above-mentioned linear regression analyses, we used 3 models adjusted for potential confounding factors, including sociodemographic, lifestyle, and medical and dietary factors, identified a priori based on established literature and expert consensus in this research field. Model 1 was adjusted for age, sex and population sample; model 2 was adjusted as model 1, plus alcohol consumption (g/d), smoking status (never, former, and current), years of education (years completed), physical activity during leisure time (a lot, moderate, little, or none), use of dietary supplements (yes/no), use of any special diet (yes/no), history of cardiovascular disease or diabetes mellitus (yes/no), family history of cardiovascular disease (yes/no), use of antihypertensive, cardiovascular disease or diabetes medication (yes/no), and for total energy intake (kcal/d) to take into account differences in metabolic efficiency as well as additional adjustment for use of potential errors in self-reported physical activity data (Supplemental Figure 3). Model 3 was adjusted as model 2, plus intake (g/1000 kcal) of low-fat dairy products, fiber-rich cereals and grains, red and processed meats, and fish and shellfish. For the linear regression analysis of individual metabolites and the metabolite score with BP and BMI only, we additionally used model 4, adjusted as model 3, plus urinary sodium excretion (mmol/24-h) as a marker of an unhealthy diet. Furthermore, we assessed whether BMI mediated the associations between the fruit and vegetable-related metabolites and metabolite score with BP (model 5). Bonferroni correction was applied to correct for multiple comparisons using a significance threshold of 3.0×10^-3^ (0.05/16). Additionally, we estimated to what extent the metabolite score-BP and BMI associations were explained by individual metabolites by adding each metabolite or urinary potassium one by one into model 3.

We repeated the analysis for 3 subcohorts, excluding participants with medical diagnoses and other traits that might bias associations between the metabolite score, BP, and BMI: *1*) excluding participants with self-reported diagnosis of hypertension or on antihypertensive treatment, *2*) excluding participants with high systolic or diastolic BP concentrations, and *3*) further exclusion of participants with a self-reported diagnosis of cardiovascular diseases or diabetes mellitus.

Statistical analyses were performed in SAS 9.4 (SAS Institute Inc.), visualizations in SIMCA 14.1 (Umetrics), MATLAB (R2021b, The MathWorks), and STATA 15 (StataCorp LLC).

## Results

### Baseline characteristics

Baseline characteristics of US and UK INTERMAP participants are presented in Supplemental Table 2. The prevalence of fruit and vegetable consumers was 99.9%; only 2 participants reported no fruit and vegetable intake at all 4 recalls. US participants had higher average fruit and vegetable intake (216 g/1000 kcal) compared with UK participants (165 g/1000 kcal). For both cohorts, fruits contributed mostly (65%) to total fruit and vegetable intake, with vegetables (24%), nuts (9%), and pulses (1%) eaten in low amounts. *Rutaceae* (22%), *Rosaceae* (13%), *Solanaceae* (12%), *Cucurbitaceae* (6%), and *Brassicaceae* (6%) were the botanical families with highest contribution to fruit and vegetable intake. High fruit and vegetable consumers presented healthier lifestyles and lower BP and BMI compared with low fruit and vegetable consumers. The reliability estimate of total fruit and vegetable intake was 72% for the total population, and 70% and 76% for the US and UK populations, respectively.

### Associations of fruit and vegetable intake with 24-h urinary ^1^H NMR spectra

**FIGURE 1:**
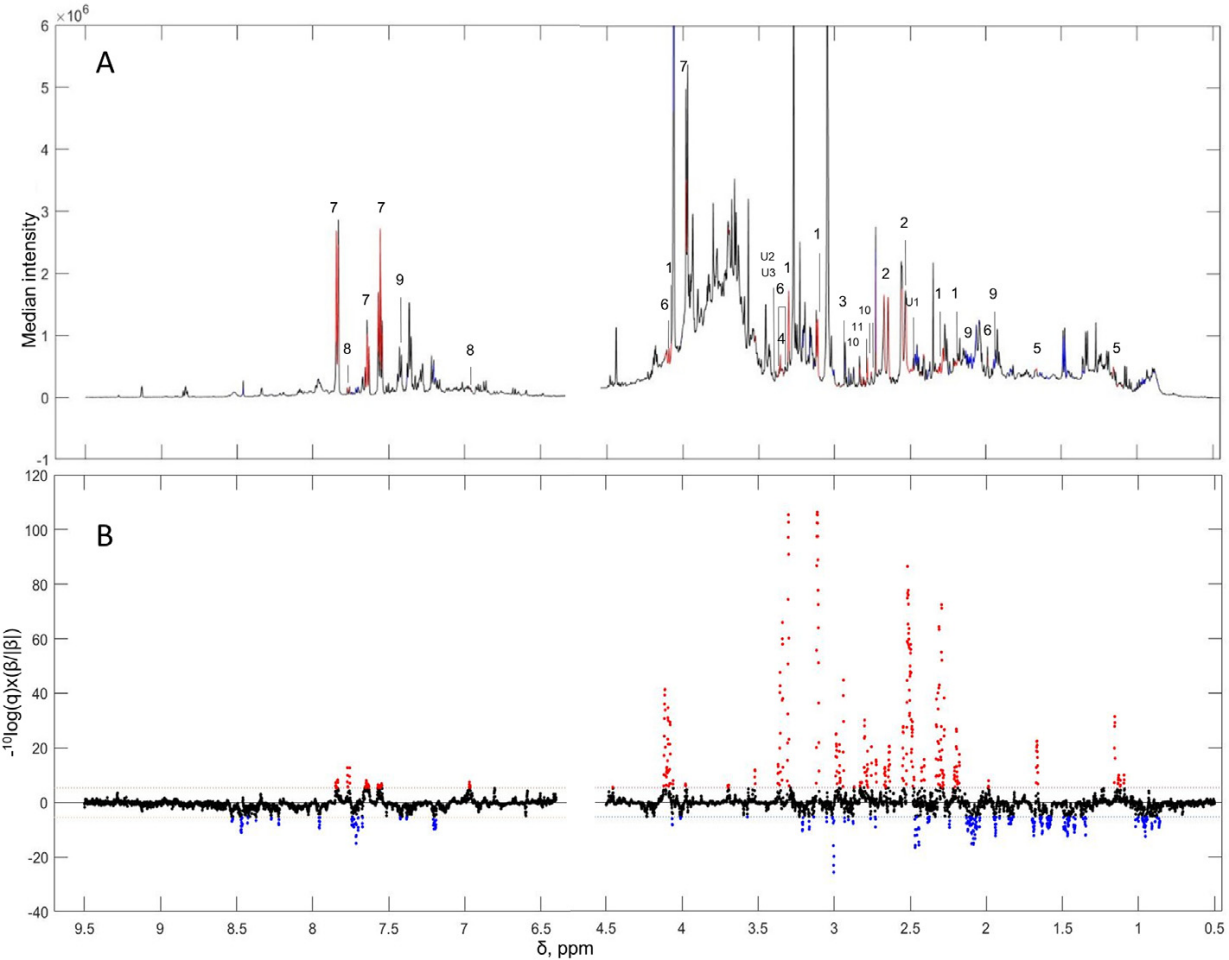
(A) Median ^1^H NMR spectrum showing significant associations of fruit and vegetable consumption with urinary metabolites using the first 24-h urinary collection of 2032 US INTERMAP participants, adjusted for age, gender, and population sample and multiple testing (*P* ≤ 4x10^-6^). (B) Manhattan plots indicating significant, positive (red), and negative (blue), spectral features (–log10(P) × sign of partial correlations). The metabolites are numbered as follows: *1*) proline betaine, *2*) Citrate, *3*) N-methylproline, *4*) scyllo-inositol, *5*) 2-hydroxy-2-(4-methyl cyclohex-3-en-1-yl) propoxyglucuronide, *6*)proline, *7*) Hippurate, *8*) 4-hydroxyhippurate, *9*) S-methyl-L-cysteine sulfoxide, *10*) S-methyl-L-cysteine sulfoxide metabolite, *11*) Phenylacetylglutamine, U1 to U3 unidentified metabolites. Details about metabolite annotation are presented in Supplemental Table 4.

Explorative orthogonal partial least-squares discriminant analysis showed discrimination of the highest compared with lowest quartiles of fruit and vegetable consumers by 7100 ^1^H NMR spectral features (Supplemental Figure 4). Sequentially, we analyzed and compared correlations between fruit and vegetable intake from 2 24-h dietary recalls in relation to 7100 ^1^H NMR spectral features from each 24-h urinary collection for each cohort separately. For the US cohort, fruit and vegetable intake was consistently associated with 402 spectral features (*P* ≤ 4×10^-6^), which corresponded to 94 spectral regions in both sets of 24-h urine samples with adjustment for age, gender, and population sample (Figure 1). Out of the 94 spectral regions, significance of 83 regions (88%) prevailed with further adjustment for lifestyle and cardiovascular disease risk factors. Of these, 48 regions (58%) were replicated in both sets of 24-h urine samples from the UK population (*P* ≤ 0.05).

### Metabolite identification

Due to the many significant ^1^H NMR spectral regions related to fruit and vegetable intake, we targeted metabolite identification on regions with the strongest correlations (*r* ≥ 0.20) in the discovery set and specific ^1^H NMR regions (0.15< *r* < 0.20) previously suggested as potential markers of fruit and vegetables (Supplemental Table 3) [9,35]. We annotated 11 metabolites, and 3 regions remained unknown (Supplemental Table 4), which comprised 55% (46 out of 83) of fruit and vegetable-related spectral regions of the US cohort. Ten annotated metabolites, namely 2-hydroxy-2-(4-methyl cyclohex-3-en-1-yl) propoxyglucuronide, 4-hydroxyhippurate, citrate, hippurate, N-methylproline, proline, proline betaine, scyllo-inositol, SMCSO, and SMCSO metabolite, were directly associated with fruit and vegetable intake whereas, phenylacetylglutamine was inversely correlated (Supplemental Table 3).

The (3-wk) ICC of these metabolites ranged from 0.30 to 0.62 and from 0.24 to 0.75 in the US and UK cohorts, respectively, and were 0.65 and 0.59 for 24-h urinary potassium excretion (Supplemental Table 5). Comparable ICCs were observed with adjustment for age, gender, and population sample.

### Associations of fruit and vegetable-related metabolites with botanical families

**FIGURE 2:**
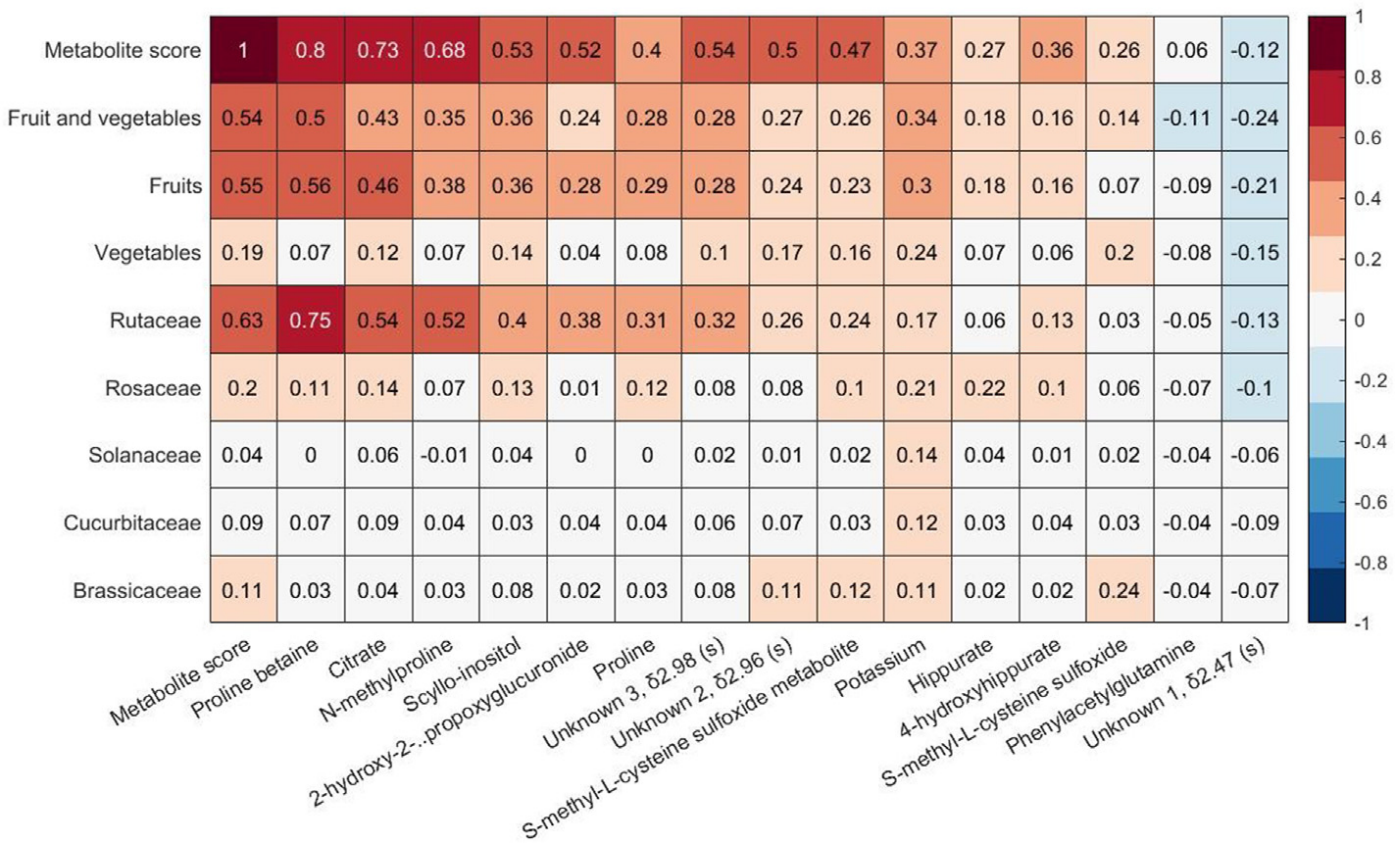
Heatmap of partial correlations between averaged fruit and vegetable intakes (total, by group and botanical family) from four 24-h dietary recalls with averaged urinary NMR-derived metabolites, potassium, and metabolic score from 2 24-h urine collections in the United States and United Kingdom INTERMAP cohorts, adjusted for age, gender, and population sample. Hierarchical clustering was used to order metabolites by correlation with fruit and vegetable consumption.

In pooled analysis, we showed that the magnitude of the correlations (*P* ≤ 4 × 10^-6^) between fruit and vegetable intake and 24-h urinary excretion of individual metabolites prevailed with further adjustments for lifestyle factors (Supplemental Table 6). The strongest correlations ranging between *r* = 0.33 to *r* = 0.49 were found for proline betaine, citrate, N-methylproline, and scyllo-inositol, which was explained by strong correlations with fruit intake, specifically *Rutaceae* (Figure 2). Hippurate and potassium showed weaker but direct correlations with *Rosaceae* intake. For vegetable intake, direct associations were found for potassium and SMCSO. Although potassium was related to a variety of vegetables, SMCSO (*r* = 0.24) and SMCSO metabolite were specifically associated with intake of *Brassicaceae*.

### Predictive value of the fruit and vegetable metabolite score

We then performed stepwise linear regression with total fruit and vegetable intake. Including all 14 fruit and vegetable-related NMR metabolites in the model achieved an *R*^*2*^ of 38.3%. With further addition of urinary potassium to the model, the *R*^*2*^ increased to 39.8%. Based on this, we created a metabolite score including the 14 fruit and vegetable-related metabolites plus urinary potassium (range: −0.87 to 2.48). A 2 SD higher metabolite score (0.77) was associated with a 139 (95% CI: 131, 148) g/1000 kcal increase in fruit and vegetable intake. The metabolite score was directly correlated with fruit and vegetable intake (*r* = 0.54), fruit (*r* = 0.55), primarily with *Rutacea* (*r* = 0.63) and *Rosacea* (*r* = 0.20), and with lower extent to vegetables (*r* = 0.19).

### Associations of individual metabolites and metabolite score with BP

**FIGURE 3:**
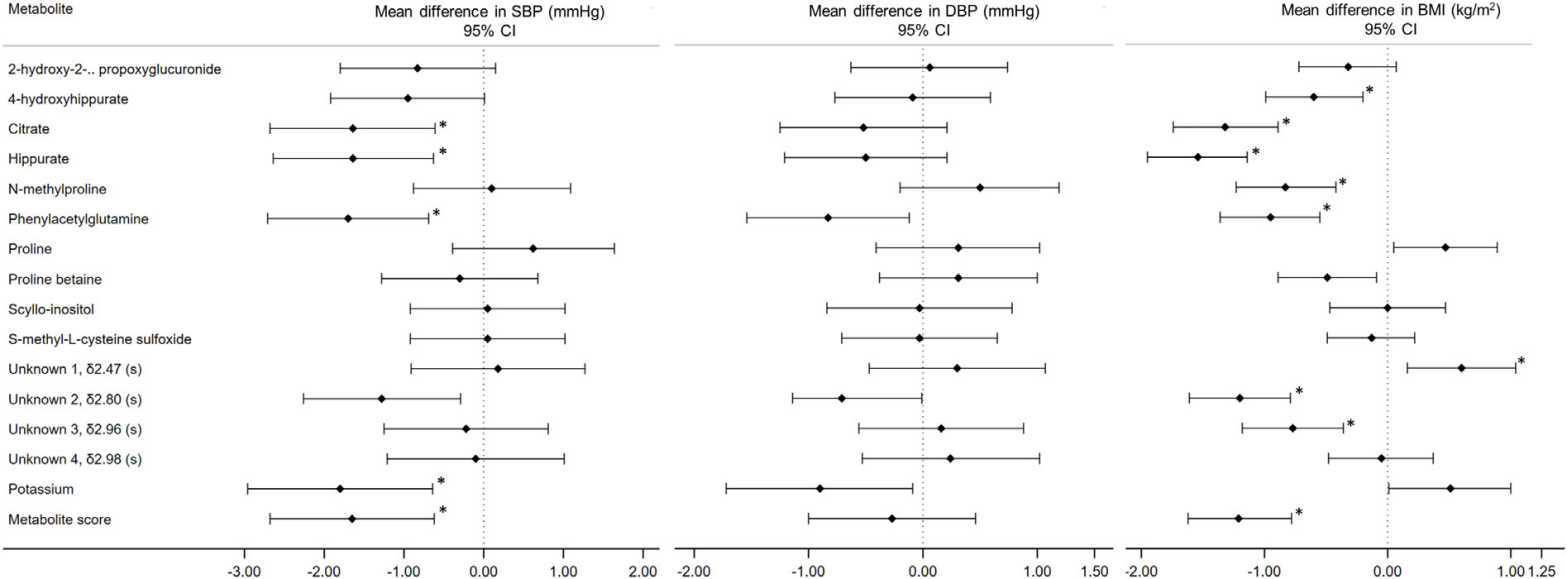
Estimated mean differences in systolic BP, diastolic BP, and BMI by 2SD of averaged urinary^1^ H NMR metabolites, metabolite score, and potassium from 2 timed 24-h urine collections in the United States and United Kingdom INTERMAP cohorts (*N* = 2481). Differences in BP and BMI and corresponding 95% confidence intervals were obtained from multivariable linear regression analyses by pooling cross-country regression coefficients weighted by sample size adjusted for age, sex, population sample, intake of energy (kcal/d), alcohol (g/d), smoking status (never, former, current), years of education (years completed), physical activity during leisure time (a lot, moderate, little or none), use of dietary supplements (yes/no), use of any special diet (yes/no), history of cardiovascular disease or diabetes mellitus (yes/no), family history of cardiovascular disease (yes/no), and use of antihypertensive, cardiovascular disease or diabetes medication (yes/no), intake (g/1000 kcal) of low-fat dairy products, fiber-rich cereals and grains, red and processed meats, and fish and shellfish, urinary sodium excretion (mmol/24-hr). BP models are additionally adjusted for BMI. Cross-country heterogeneity of regression coefficients was assessed by chi-square test: no significant heterogeneity across countries was detected. A Bonferroni correction was applied, resulting in a significance level of *P* ≤ 3.0 × 10^-3^; ^∗^ indicates significant associations.

After extensive adjustments, including dietary factors and BMI and correction for multiple testing, a 2SD higher metabolite score was associated with a −1.65 mmHg lower systolic BP (95% CI: −2.68, −0.62; *P* < 0.002). This was mainly explained by significant systolic BP associations with citrate and hippurate, as well as phenylacetylglutamine and urinary potassium excretion (Figure 3, Supplemental Table 7). The association between the metabolite score and systolic BP attenuated with additional adjustment of citrate, whereas stronger associations were observed with additional adjustment for proline betaine, N-methylproline, and proline (Supplemental Table 8). In sensitivity analyses, associations between the metabolite score and systolic BP were comparable in subcohorts free of hypertension, diabetes, and cardiovascular disease (Supplemental Table 9). The metabolite score and individual fruit and vegetable-related metabolites were not associated with diastolic BP (Figure 3, Supplemental Table 7).

### Associations of individual metabolites and metabolite score with BMI

The metabolite score was associated with a −1.21 kg/m^2^ lower BMI (Supplemental Table 7, by 2SD; 95% CI: −1.62, −0.78; *P* < 0.0001). This was explained by significant inverse associations with BMI for citrate, hippurate, N-methylproline, SMCSO metabolite, and phenylacetylglutamine. Urinary potassium, however, was directly associated with BMI (+1.30, 95% CI: 0.82, 1.77; Figure 3, Supplemental Table 7). The significant metabolite score-BMI association attenuated with additional adjustment for citrate, whereas stronger associations were observed with adjustment for proline and urinary potassium (Supplemental Table 8). The metabolite score-BMI association remained significant in sensitivity analysis in subcohorts free of hypertension, diabetes, and cardiovascular diseases (Supplemental Table 9).

## Discussion

We have characterized here 11 NMR-derived 24-h urinary metabolites related to fruit and vegetable consumption. We found the metabolites to be consistent and reproducible across multiple 24-h urine collections and 2 populations. Of the 11 identified metabolites, 9 metabolites were associated with fruit intake, many of them specifically with *Rutaceae* intake, and 3 metabolites showed weaker but significant associations with vegetables, specifically *Brassicaceae*. We built a metabolite score comprising all identified metabolites plus urinary potassium predictive of fruit and vegetable intake, which showed strong inverse associations with systolic BP and BMI. For both cardiovascular disease risk factors, these associations were, to a large extent, explained by citrate.

In the past decades, highly controlled feeding studies have discovered many metabolites that may serve as candidate markers of fruit and vegetable intake beyond established biomarkers [9,10,36]. The utility of these metabolites in free-living populations, however, depends on eating behaviors with variability in intake levels, frequencies, time-dependent responses following intake, and a variety of compounds across fruit and vegetable types [37]. We confirmed a set of urinary NMR-derived metabolites (proline betaine, citrate, N-methylproline, scyllo-inositol, and 2-hydroxy-2-[4-methyl cyclohex-3-en-1-yl] propoxyglucuronide) that showed strong direct associations with *Rutacea*e intake. In our population-based cohorts, *Rutacea*e (22%) had the highest contribution to fruit and vegetable intake [38]. Consequently, it is not surprising that we found that ∼50% of the identified metabolites were related to *Rutacea*e intake. We further confirmed SMCSO as marker of *Brassicaceae* and hippurate, a gut microbial cometabolite, as marker of *Rosaceae* in these populations. 4-hydroxyhippurate and potassium showed weaker associations with multiple botanical families, explained by their excretions following intake of a wide range of plant-based food sources beyond fruit and vegetables. These findings are in line with results from small-scale highly controlled feeding trials [9,10,36] and previous reports on metabolomics to assess populations’ adherence to healthful dietary patterns [11,12,26] and strengthens the potential of these metabolites as biomarkers of fruit and vegetable intake. In addition, we showed that fruit and vegetable-related metabolites were reproducible and consistent across repeated 24-h urinary collections and populations and persisted after adjustment for potential confounders. These findings underpin the potential of these metabolites as robust biomarkers of fruit and vegetable intake in free-living populations with substantial variability in eating behaviors.

The fruit and vegetable metabolite score allowed us to assess the predictive value of this combination of metabolites and if prediction could be improved with the addition of urinary potassium. Compared with using a single metabolite, we found that the combined metabolite score was able to predict fruit and vegetable intake as previously reported, with a combined marker of serum carotenoids and plasma vitamin C in various fruit and vegetable intervention studies [39]. Specifically, our urinary NMR-derived metabolite score achieved a higher correlation with fruit and vegetable intake (*r* = 0.54) compared with previously reported scores based on circulating carotenoids and vitamin C in the UK-based EPIC-Norfolk cohort (*r* = 0.42) [40] and the US National Health and Nutrition Examination Survey III (*r* = 0.28) [41]. We found that adding urinary potassium to the model only slightly improved the predictive value, which may be explained by overlap of fruit and vegetable sources of potassium and the urinary metabolites. However, using combined scores to fully capture the biochemically complex food group of fruits and vegetables needs a wide range of biomarkers assessed from different biofluids and analytical platforms with various sensitivities with validation in different populations calls for further investigation.

Our findings are consistent with results from a meta-analysis of 18 prospective cohort studies, which indicated that higher fruit and vegetable intake was associated with a 9%–11% lower risk of hypertension [42], and with results from landmark dietary interventions that demonstrated beneficial systolic BP-lowering effects of high fruits and vegetable intake [43,44]. Our study showed a robust inverse association between the metabolite score and systolic BP that persisted even after adjustment with BMI, which was mainly explained by citrate and SMCSO metabolite. Citrate, naturally occurring in citrus fruits and often used as a food additive [45], is a tricarboxylic acid intermediate involved in the homeostatic regulation of calcium and magnesium [46]. Although limited evidence is available on the impact of citrate on BP, our findings align with previous research indicating inverse associations of citrate with atherosclerosis [47], and incident cardiovascular diseases [47,48], suggesting potential BP-lowering benefits of citrate. S-methyl cysteine sulfoxide metabolite is a sulfur-containing compound and well-known marker of cruciferous vegetables [10]. Sulfur-containing compounds are essential for regulating endothelial function; deficiency in sulfur contributes to endothelial dysfunction, a precursor of atherosclerosis [49]. In addition, we observed that the strength of the inverse association of the metabolite score with systolic BP substantially improved when proline betaine, N-methylproline, or proline were added to the model, suggesting potential effect modification by citrus fruit intake. Although associations between these individual metabolites and BP were not significant in our cross-sectional study, results from the Dietary Approaches to Stop Hypertension (DASH)-Sodium trial showed that urinary excretion of these metabolites was significantly associated with a reduction of systolic and diastolic BP in those consuming a DASH diet but not a control diet [50]. How these metabolites may be involved in underlying mechanisms by which fruit and vegetables may influence BP needs further investigation.

Relative to BMI, we found inverse associations with the metabolite score, which are in line with results of meta-analysis of 8 dietary interventions [51] and 17 cohort studies [52] that showed that fruit and vegetable intake can contribute to maintenance of body weight. Also, in our research, greater fruit and vegetable intake was accompanied by higher dietary fiber intakes. Research is well-established on the prolonged digestive effects of dietary fiber, leading to increased satiety and, consequently, reduced food intake and body weight [53]. The inverse associations of hippurate and phenylacetylglutamine with BMI confirm the potential mechanism involving delayed digestion following fruit and vegetable intake. This aligns with findings from previous research indicating that higher fruit and vegetable intake is related to higher circulating concentrations of hippurate and lower phenylacetylglutamine, which serve as markers of gut microbial diversity [54]. In addition, we found weaker but significant inverse associations between N-methylproline and proline betaine, both markers of citrus fruit, with BMI. There is only limited research on citrus fruit and body weight, with evidence on the effects of citrus fruit intake and energy metabolism mainly based on orange juice. However, research findings have accumulated evidence for their effects on energy metabolism and lipid oxidation by increased thermogenesis and mitochondrial biogenesis [55] that may explain our findings. The observed metabolites associated with BMI could be merely indicators of higher intakes of fruits and vegetables with a high biochemical complexity. The underlying mechanisms by which fruits and vegetables may influence body weight warrant further investigation.

The main strength of our study includes the use of untargeted ^1^H NMR urinary metabolomic profiles from 2 timed 24-h urinary collections with corresponding repeated detailed 24-h dietary recalls in 2 population samples, facilitating discovery of potential urinary biomarkers of fruit and vegetable intake and minimizing within-person variability of diet. Standardized methods were used for investigation of diet, BP, BMI, ^1^H NMR, and urinary potassium and sodium measurements across population samples, limiting measurement bias. The high reproducibility and intraclass correlations of identified urinary features over time and across populations between the replication and validation sets suggest robustness of identified markers of fruit and vegetable intake. This allowed standardized and objective investigation of associations between fruit and vegetable intake, BP, and BMI across 2 Western population samples. Limitations include the cross-sectional nature of this study, suggesting that our findings require confirmation from longitudinal and experimental research. We applied a higher false discovery rate for the smaller sample size of the UK cohort, but this may have limited validation of some metabolic features. The untargeted metabolic profiling approach allowed discovery of metabolic features; however, we were not able to annotate or identify all metabolic features. Although models were extensively adjusted for intake of energy and other lifestyle and dietary factors, we cannot rule out potential of residual confounding. Finally, we used self-reported data on fruit and vegetable intakes, which is subject to measurement error and recall bias. This, combined with the wide range of reported fruit and vegetable types and their biochemical complexities, suggests the need for replication in controlled dietary intervention studies to examine potential underlying pathways through which fruit and vegetables may influence BP and body weight.

Taken together, our analysis showed the potential of NMR-derived metabolites to serve as biomarkers to objectively assess fruit and vegetable intake in population-based cohorts. The fruit and vegetable-related metabolite score was inversely associated with systolic BP and BMI, which was mainly explained by citrate. Additional observational and metabolomic studies are needed to validate our findings and to elucidate the mechanisms underlying the health benefits of fruit and vegetables.

## Author contributions

The authors’ contributions were as follows – LVH, PE, JS: designed the INTERMAP study, conducted the fieldwork, and collected data; LOG: performed dietary coding and dietary data analysis, statistical analysis, interpreted the data, and drafted the manuscript; EC, LOG: performed the biochemical analyses and metabolite identification; EC, LVH, QC, MLD, GF, EH, TE, PE: revised the work critically for important intellectual content; and all authors: read and approved the final manuscript.

## Funding

The INTERMAP Study was supported by grants (R01-HL50490, R01-HL65461, R01-HL84228, and R01-HL135486) from the National Heart, Lung, and Blood Institute, National Institutes of Health (Bethesda, Maryland, United States) also by the national agencies in the United Kingdom (project grant from the West Midlands National Health Service Research and Development, and grant R2019EPH from the Chest, Heart and Stroke Association, Northern Ireland). Infrastructure support was provided by the National Institute for Health Research (NIHR) Imperial Biomedical Research Center (BRC).

These analyses were conducted with financial support to LOG from the Imperial College London Junior Research Fellowship (F24074), Academy of Medical Sciences Springboard award (SBF003\1101), and the NIHR Cambridge BRC (NIHR203312). PE is Director of the MRC Centre for Environment and Health (MRC/S019669/1). The sponsors had no role in the design or conduct of the study, the collection, management, analysis, or interpretation of the data, or the preparation, review, or approval of the manuscript. The views expressed are those of the authors and not necessarily those of the NIHR or the Department of Health and Social Care.

### Data sharing

An anonymized data set, including data described in the manuscript, code book, and analytic code, is available upon request pending application and approval.

### Study registration

This study was registered at the clinicaltrials.gov registration (https://clinicaltrials.gov/study/NCT00005271?term=NCT00005271&rank=1) as NCT00005271.

## Conflict of interest

QC is an employee and shareholder of Amgen Inc. The work presented here was conducted while QC was an employee of Imperial College London. Amgen Inc. was not involved in this study. All other authors declare no competing interests.
