# Supplementary material for: A Metabolome Wide Association Study of Fruit and Vegetable Consumption and Associations with Cardiovascular Disease Risk Factors: The International Study of Macro-/Micronutrients and Blood Pressure (INTERMAP) Study
Source: J Nutr. 2024 Nov 12;155(1):122–31. doi: 10.1016/j.tjnut.2024.11.004 (PMC11795696; doi:10.1016/j.tjnut.2024.11.004)
Supplement: Multimedia component 1 [file mmc1.docx]

**Supplementary Material**

# **Title: A Metabolome-Wide Association Study of fruit and vegetable intake and associations with cardiovascular risk factors: the INTERMAP study**

**Authors:** Linda M. Oude Griep, Elena Chekmeneva, Linda Van Horn, Queenie Chan, Martha L. Daviglus, Gary Frost, Elaine Holmes, Tim Ebbels, and Paul Elliott

**Correspondence**: Dr Linda M. Oude Griep, Email: linda.oudegriep@mrc-epid.cam.ac.uk

## Supplemental methods

**Exclusion criteria**

Of 4,895 participants surveyed, individuals were excluded who did not attend all four visits (n=110), with incomplete/missing data or 24-hr urine sample (n=61), provided unreliable dietary data (n=7), or reported extreme total energy intake (<500 or > 5000 kcal/day for women and < 500 or >8000 kcal/day for men, n=37). Hence, 4680 participant (2,359 men and 2,321 women) were included in the INTERMAP study. Previous findings showed significantly heterogeneous urinary metabolic phenotypes of East Asian participants compared to Western participants[1], we therefore focus here on the Western population (n=2,696). We further excluded Western participants with missing ^1^H NMR spectroscopy data (n= 36) and metabolic outliers (n=179); data of 2,481 participants (2,032 US and 449 UK individuals) remained for this analyses (**Figure S2**).

Metabolic outliers were identified using the Hotelling’s T^2^ statistic (95% criterion) on the scores of Principal Component Analysis. Metabolic outliers were defined as participants whose scores, for either urine collection, mapped outside the Hotellings T^2^ ellipse with confidence interval of 95% in a cross-validated seven-component model.[1] Participants who were identified as metabolic outliers often showed high ethanol or nonsteroidal anti-inflammatory drug excretion.[1]

## Supplemental Figure 1. Schematic design INTERMAP study showing data collection periods of 24-hr dietary recalls and urine collections


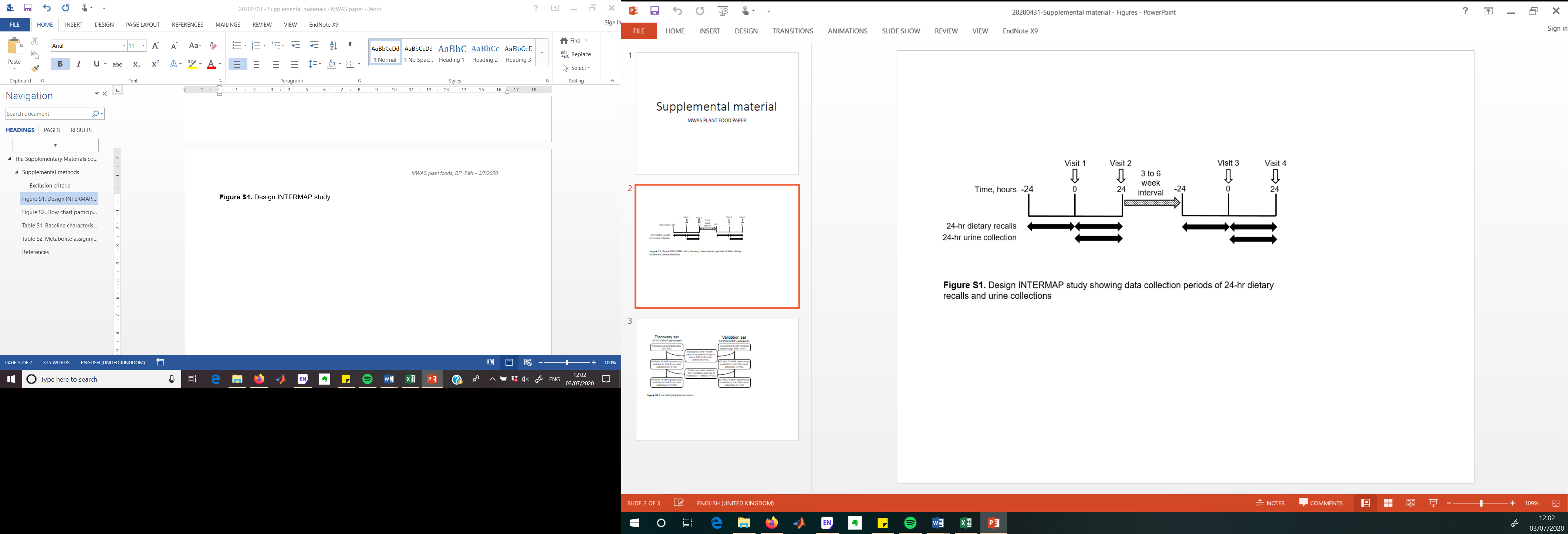


## Supplemental Figure 2. Participant flow chart for the US (discovery) and UK (validation) INTERMAP cohorts


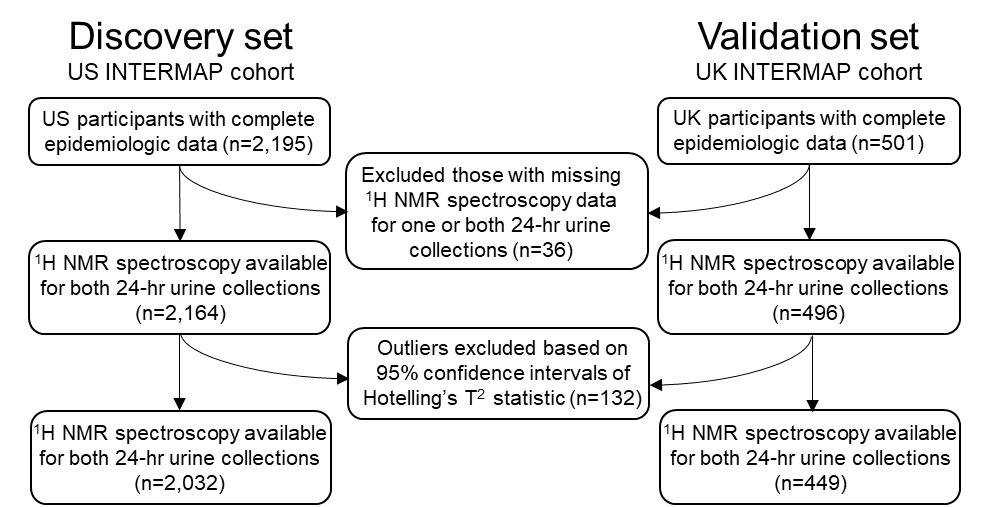


## Supplemental Figure 3. Directed acyclic graph of relationships of included confounding factors with the metabolite score and blood pressure [2]


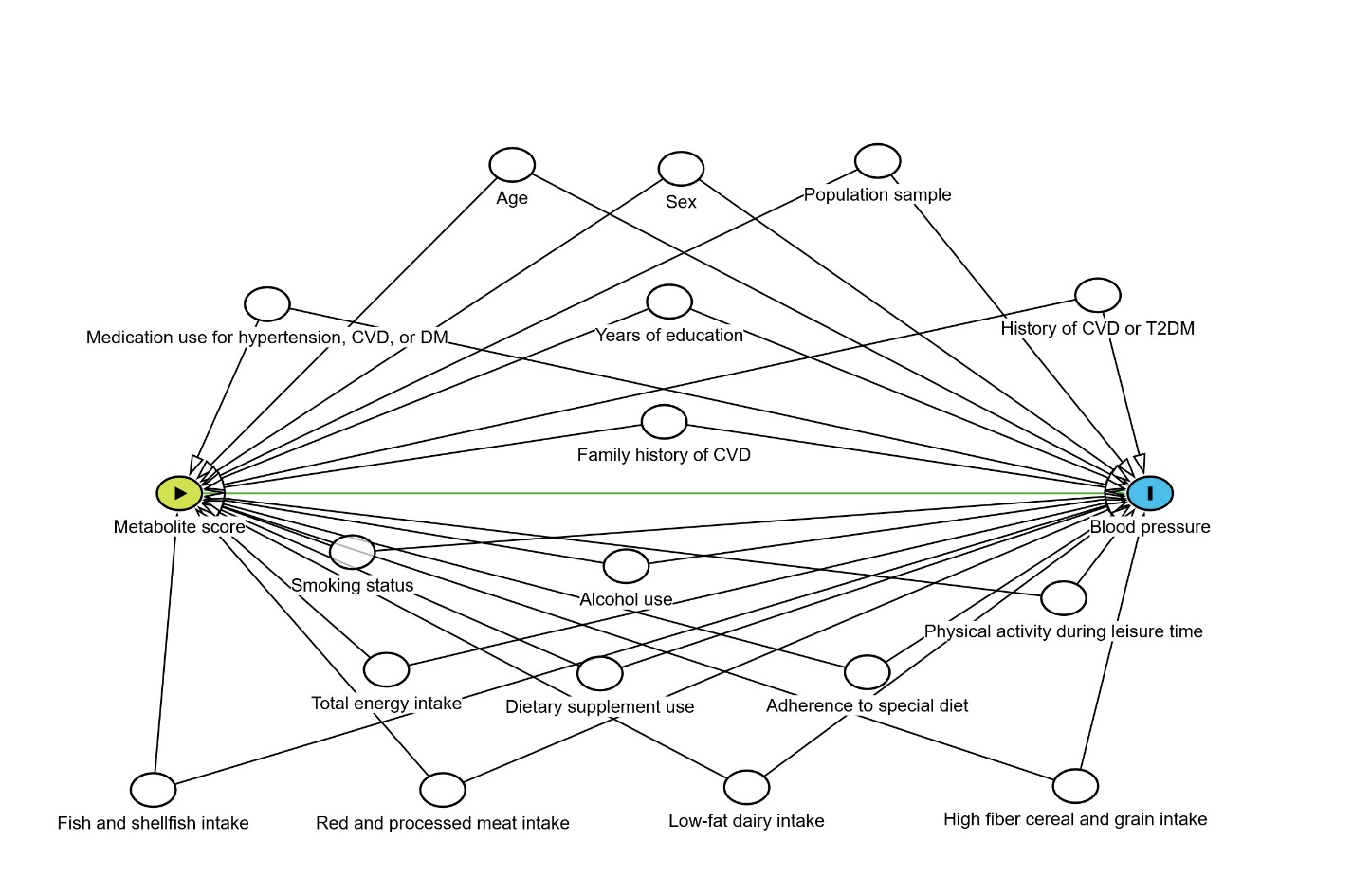


## Supplemental Table 1. Overview of botanical families and most commonly consumed fruit and vegetables reported among the US and UK INTERMAP cohorts

| Botanical family | Individual fruits or vegetables |
| --- | --- |
| Rutaceae | Grapefruit, kumquat, lemon, lime, orange, pomelo, satsuma, tangerine, citrus fruit undefined |
| Rosaceae | Almond, apple, apricot, blackberry, cherry, hawthorn, loquat, nectarine, peach, pear, plum, prune, raspberry, strawberry |
| Solanaceae | Chilli pepper, sweet pepper, tomato |
| Cucurbiceae | Courgette, cucumber, eggplant, gherkin, melon, pumpkin, squash |
| Brassicaceae | Broccoli, Brussels sprouts, cabbage, cauliflower, kale, other leafy vegetables, radish, sauerkraut, turnip, watercress |

## Supplemental Table 2. Baseline characteristics of the US and UK INTERMAP cohorts by median fruit and vegetable intake and for the total cohort^1^

|  | US (N=2,032) | | UK (N=449) | | US+UK cohort (N=2,481) |
| --- | --- | --- | --- | --- | --- |
|  | Lower^2^ | Higher^2^ | Lower^2^ | Higher^2^ |  |
| Age, y | 48.3 ±5.4 | 49.9 ±5.2 | 48.5±5.6 | 49.7±5.5 | 49.1±5.4 |
| Men, % | 57.8 | 44.6 | 61.6 | 48.4 | 51.9 |
| **Lifestyle factors** |  |  |  |  |  |
| Education, y | 14.6±3.0 | 15.5±2.7 | 12.6±2.7 | 12.9±3.5 | 14.6±3.2 |
| Physically active during leisure time^3^, % | 57.4 | 67.1 | 54.9 | 52.0 | 60.7 |
| Current smoker, % | 22.7 | 8.6 | 24.6 | 9.8 | 15.8 |
| Alcohol use, g/d | 7.4±14.0 | 5.3±9.4 | 15.7±18.4 | 13.7±19.2 | 7.9±13.9 |
| Dietary supplement use, % | 44.8 | 60.0 | 29.5 | 41.8 | 49.4 |
| Adherence to special diet, % | 11.5 | 23.9 | 11.6 | 28.4 | 18.1 |
| **Cardiovascular risk factors** |  |  |  |  |  |
| Systolic blood pressure, mmHg | 119.7±13.4 | 116.7±13.8 | 120.1±14.3 | 120.5±14.3 | 118.6±13.8 |
| Diastolic blood pressure, mmHg | 74.2±9.8 | 72.5±9.5 | 77.5±9.1 | 77.5±10.4 | 74.1±9.8 |
| Body Mass Index, kg/m^2^ | 29.6±5.8 | 28.1±5.8 | 27.6±5.1 | 27.2±4.0 | 28.6±5.6 |
| Family history of high BP | 67.6 | 67.6 | 46.4 | 50.2 | 64.1 |
| History of CVD or DM, % | 14.6 | 14.0 | 12.5 | 7.6 | 13.5 |
| Use of medication for hypertension, CVD, or DM, % | 23.9 | 23.4 | 11.2 | 20.9 | 22.3 |
| **Urinary electolyte excretions^4^** | |  |  |  |  |
| Potassium, mmol/24 hr | 53.3±19.1 | 62.9±21.6 | 63.4±20.2 | 74.0±20.2 | 60.1±21.1 |
| Sodium, mmol/24 hr | 170.2±60.3 | 157.0±56.7 | 149.1±46.2 | 142.2±46.2 | 160.3±57.6 |
| Sodium to potassium, ratio | 3.4±1.3 | 2.7±1.0 | 2.5±0.8 | 2.0±0.8 | 2.9±1.2 |
| **Food group intakes, g/1000 kcal^5^** |  |  |  |  |  |
| Fruit | 65±38 | 218±107 | 43±98 | 169±98 | 135±109 |
| Vegetables | 36±23 | 69±48 | 23±33 | 53±33 | 49±40 |
| Pulses | 15±17 | 22±25 | 17±25 | 21±25 | 18±22 |
| Nuts | 3±5 | 3±5 | 1±4 | 1±4 | 2±4 |
| Botanical families |  |  |  |  |  |
| *Rutaceae* | 17±26 | 79±82 | 13±20 | 54±60 | 45±65 |
| *Rosaceae* | 10±1 | 40±47 | 13±17 | 48±46 | 26±38 |
| *Solanaceae* | 19±17 | 33±25 | 8±10 | 26±28 | 24±26 |
| *Cucurbitaceae* | 5±13 | 24±43 | 1±3 | 11±32 | 13±32 |
| *Brassicaceae* | 7±15 | 16±29 | 8±19 | 23±32 | 12±24 |
| Low-fat dairy | 37±78 | 65±94 | 23±81 | 54±81 | 49±84 |
| Fiber-rich cereals and grains | 12±14 | 16±17 | 20±42 | 44±42 | 17±22 |
| Fish and shellfish | 8±13 | 11±17 | 10±16 | 12±16 | 10±15 |
| Meat | 150±79 | 112±67 | 127±71 | 108±67 | 129±75 |
| **Nutrient intakes^5^** |  |  |  |  |  |
| Total energy, kcal | 2394±721 | 2095±638 | 2318±641 | 2047±571 | 2233±684 |
| Total Protein, % kcal | 15±3 | 16±3 | 15±3 | 16±3 | 16±3 |
| Animal protein, % kcal | 10±3 | 10±3 | 10±3 | 10±3 | 10±3 |
| Vegetable protein, % kcal | 5±1 | 6±2 | 6±1 | 7±1 | 5±2 |
| Total carbohydrates, % kcal | 48±8 | 52±8 | 43±6 | 46±7 | 49±8 |
| Dietary fibre, g/day | 17±7 | 21±9 | 23±8 | 28±9 | 20±9 |
| Total sugars, % kcal | 25±8 | 28±8 | 19±5 | 22±6 | 26±8 |
| Starch, % kcal | 22±5 | 24±6 | 25±5 | 26±5 | 23±6 |
| Total Fat, % kcal | 35±6 | 31±7 | 35±6 | 30±6 | 33±7 |
| Mono unsaturated fatty acids (MUFA), % kcal | 13±3 | 11±3 | 12±2 | 10±2 | 12±3 |
| Poly unsaturated fatty acids (PUFA), % kcal | 7±2 | 7±2 | 7±2 | 6±2 | 7±2 |
| Saturated fatty acids (SFA), % kcal | 12±3 | 10±3 | 13±3 | 11±3 | 11±3 |
| Trans-fatty acids, % kcal | 2±1 | 2±1 | 2±1 | 1±1 | 2±1 |
| Cholesterol, mg/1000kcal | 141±58 | 120±56 | 123±46 | 116±48 | 128±57 |
| Vitamin C, mg/1000kcal | 32±17 | 75±36 | 26±12 | 60±30 | 52±35 |
| Potassium, mg/1000kcal | 1172±257 | 1552±351 | 1439±248 | 1812±357 | 1410±375 |
| Beta-carotene, mcg/1000kcal | 1297±1255 | 2601±2294 | 732±539 | 1475±1004 | 1796±1843 |

| ^1^ Mean±SD (all such values)  ^2^ Cut-off lower/higher total fruit and vegetable intake for US cohort: 188.9, for UK cohort 143.1 g/1000 kcal |
| --- |
| ^3^ Defined as engagement in moderate or heavy physical activity during leisure time |
| ^4^ Average of 2 timed 24-hr urine collections |
| ^5^ Average of 4 multiple pass 24-hr dietary recalls |

##
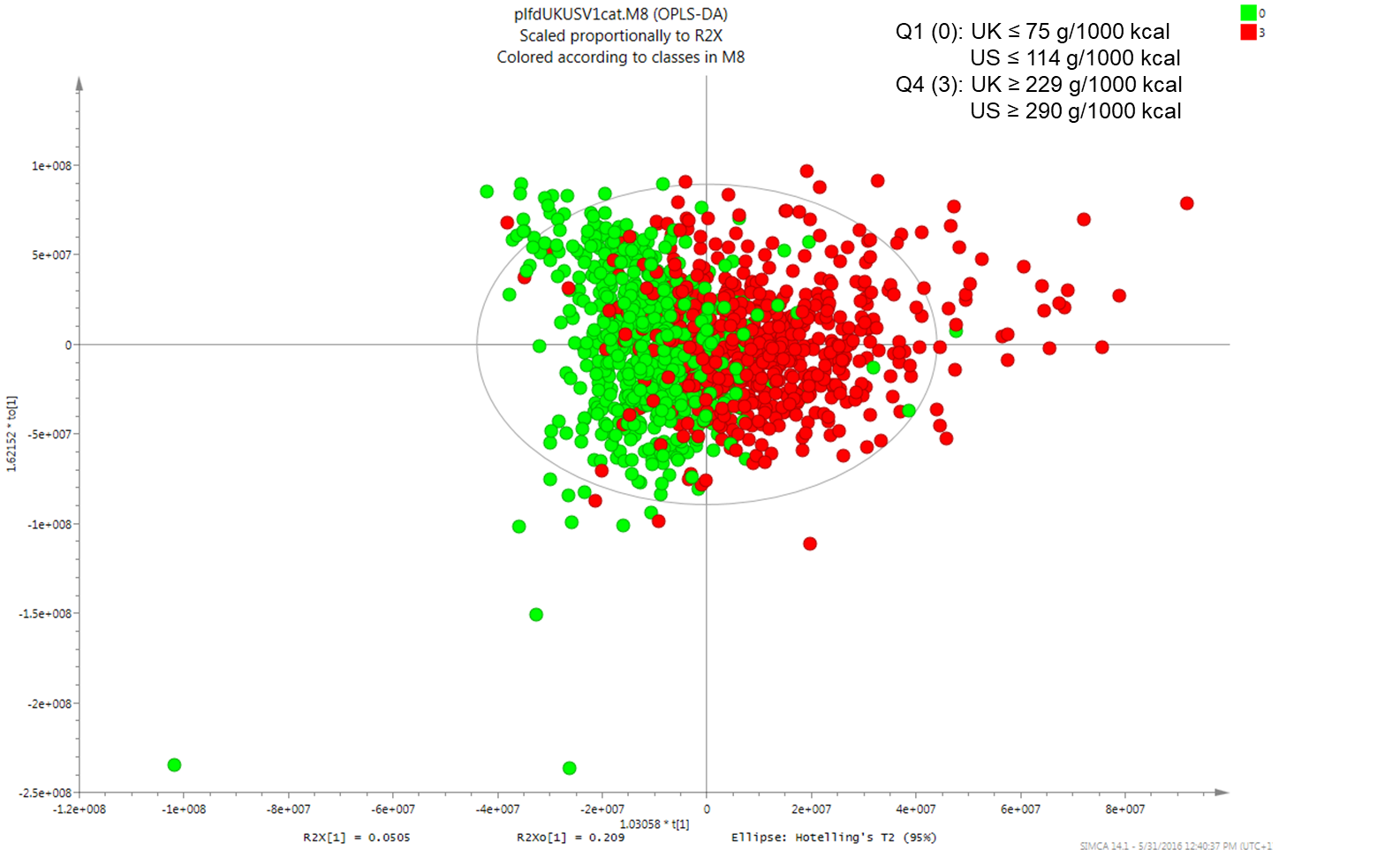
Supplemental Figure 4. Results from orthogonal partial least-squares discriminant analysis (OPLS-DA) showing the discrimination of highest vs lowest country-specific quartiles of fruit and vegetable consumers by 7,100 1H NMR spectral data points. Green circles represent the quartile 1 and red circles represent quartile 4. X-axis, first predictive component explaining the variation between groups; Y-axis, second predictive component explaining the variation within groups.

## Supplemental Table 3. Partial correlations of individual ^1^H NMR features with smallest P-value associated with fruit and vegetable intake assessed by 2 24-hr dietary recalls for each visit of US and UK INTERMAP cohorts^1^

|  |  |  | US (N=2,032) | | | | UK (N=449) | | | | | |
| --- | --- | --- | --- | --- | --- | --- | --- | --- | --- | --- | --- | --- |
|  |  |  | Visit 1 | | Visit 2 | | Visit 1 | | | Visit 2 | | |
|  | PPM with smallest P | PPM range | R | P-value | R | P-value | R | P-value | | R | | P-value |
| 2-hydroxy-2-(4-methyl cyclohex-3-en-1-yl) propoxyglucuronide | 1.1565 | 1.1555-1.1585 | 0.27 | 4.8x10^-34^ | 0.20 | 3.7x10^-20^ | 0.15 | 1.0x10^-3^ | 0.03 | | 5.2x10^-1^ | |
| 4-hydroxyhippurate | 7.7565 | 7.7565 | 0.17 | 6.1x10^-15^ | 0.24 | 6.2x10^-25^ | 0.11 | 2.0x10^-2^ | 0.11 | | 1.5x10^-2^ | |
| Citrate | 2.5185 | 2.4845-2.5255 | 0.43 | 6.6x10^-90^ | 0.40 | 2.2x10^-77^ | 0.33 | 5.5x10^-13^ | 0.31 | | 2.1x10^-11^ | |
| Hippurate | 7.8355 | 7.8335-7.8385 | 0.14 | 2.7x10^-10^ | 0.17 | 7.1x10^-14^ | 0.22 | 2.3x10^-6^ | 0.25 | | 5.3x10^-8^ | |
| N-methylproline | 2.9395 | 2.9385-2.9405 | 0.31 | 1.3x10^-47^ | 0.32 | 6.3x10^-50^ | 0.25 | 8.4x10^-8^ | 0.32 | | 3.1x10^-12^ | |
| Phenylacetylglutamine | 2.0975 | 2.0955-2.1025 | -0.19 | 1.5x10^-17^ | -0.22 | 1.2x10^-23^ | -0.25 | 6.1x10^-8^ | -0.21 | | 7.3x10^-6^ | |
| Proline | 4.1125 | 4.1115-4.1175 | 0.30 | 3.9x10^-44^ | 0.21 | 9.3x10^-22^ | 0.31 | 1.1x10^-11^ | 0.30 | | 5.4x10^-11^ | |
| Proline betaine | 3.1115 | 3.1015-3.1175 | 0.47 | 6.5x10^-111^ | 0.42 | 3.1x10^-87^ | 0.46 | 1.1x10^-24^ | 0.47 | | 1.3x10^-25^ | |
| Scyllo-inositol | 3.3565 | 3.3545-3.3575 | 0.32 | 1.9x10^-50^ | 0.26 | 3.1x10^-32^ | 0.25 | 1.1x10^-7^ | 0.25 | | 7.8x10^-8^ | |
| S-methyl-L-cysteine sulfoxide | 2.7555 | 2.7555 | 0.22 | 8.6x10^-23^ | 0.26 | 1.6x10^-32^ | 0.20 | 1.3x10^-5^ | 0.10 | | 3.9x10^-2^ | |
| S-methyl-L-cysteine sulfoxide metabolite | 2.8005 | 2.7995-2.8035 | 0.26 | 1.1x10^-32^ | 0.22 | 3.8x10^-23^ | 0.17 | 3.1x10^-4^ | 0.19 | | 1.2x10^-3^ | |
| Unknown 1 (δ2.47) | 2.4685 | 2.4675-2.4695 | -0.20 | 6.1x10^-19^ | -0.22 | 3.4x10^-24^ | -0.23 | 1.3x10^-6^ | -0.16 | | 5.7x10^-4^ | |
| Unknown 2 (δ2.96) | 2.9655 | 2.9605-2.9675 | 0.22 | 1.4x10^-23^ | 0.24 | 1.1x10^-28^ | 0.12 | 1.5x10^-2^ | 0.23 | | 1.5x10^-6^ | |
| Unknown 3 (δ2.98) | 2.9875 | 2.9825-2.9895 | 0.24 | 1.2x10^-27^ | 0.26 | 2.9x10^-33^ | 0.23 | 1.4x10^-6^ | 0.20 | | 1.5x10^-5^ | |

**^1^** Statistical significance was at the metabolome-wide significance level (P≤4x10^-6^) for the US cohort and at the False Discovery Rate (P≤0.05) for the UK cohort. Analyses were adjusted for age, sex, and population sample.

## Supplemental Table 4. Details of annotation and identification of ^1^H NMR metabolites associated with fruit and vegetable intake

| \| Metabolite \| ^1^H ppm^1^ \| Level of Annotation^2^ \| Method(s) of confirmation \| \| --- \| --- \| --- \| --- \| \| 2-hydroxy-2-(4-methyl cyclohex-3-en-1-yl) propoxyglucuronide \| 1.16 (s), 1.67 (s) \| 2 \| STOCSY, STORM \| \| 4-hydroxyhippurate \| 6.97 (d), 7.76 (d) \| 2 \| STOCSY, STORM, 2D NMR \| \| Citrate \| 2.52 (d), 2.66 (d) \| 1 \| STOCSY, STORM, 2D NMR \| \| Hippurate \| 3.98 (d), 7.55 (t), 7.64 (t), 7.84 (d) \| 1 \| STOCSY, STORM, 2D NMR \| \| N-methylproline \| 2.94 (s) \| 1 \| STOCSY, STORM, 2D NMR, ^1^H NMR spike \| \| Phenylacetylglutamine \| 1.94 (m), 2.11 (m), 7.43 (t) \| 1 \| STOCSY, STORM, 2D NMR, ^1^H NMR spike \| \| Proline \| 1.99 (m), 3.34 (dt), 3.37 (dt), 4.11 (dd) \| 1 \| STOCSY, STORM, 2D NMR, ^1^H NMR spike \| \| Proline betaine \| 2.18 (m), 2.30 (m), 3.11 (s), 3.30 (s), 4.09 (m) \| 1 \| STOCSY, STORM, 2D NMR, ^1^H NMR spike \| \| Scyllo-inositol \| 3.36 (s) \| 1 \| STOCSY, STORM, 2D NMR, ^1^H NMR spike \| \| S-methyl-L-cysteine sulfoxide \| 2.75 (s), 2.78 (s), 2.83 (s) \| 2 \| STOCSY, STORM, ^1^H NMR spike \| \| S-methyl-L-cysteine sulfoxide metabolite \| 2.80 (s) \| 2 \| STOCSY, STORM \| \| Unknown 1 \| 2.47 (s) \| 4 \| STOCSY, STORM \| \| Unknown 2 \| 2.96 (s) \| 4 \| STOCSY, STORM \| \| Unknown 3 \| 2.98 (s) \| 4 \| STOCSY, STORM \| |
| --- | --- | --- | --- | --- | --- | --- | --- | --- | --- | --- | --- | --- | --- | --- | --- | --- | --- | --- | --- | --- | --- | --- | --- | --- | --- | --- | --- | --- | --- | --- | --- | --- | --- | --- | --- | --- | --- | --- | --- | --- | --- | --- | --- | --- | --- | --- | --- | --- | --- | --- | --- | --- | --- | --- | --- | --- | --- | --- | --- | --- |

Multiplicity: s=singlet, d=doublet, t=triplet, m=multiplet, dd=doublet of doublets, dt=doublet of triplets

^2^Levels of confidence in the assignment of the identified metabolites according to the Metabolomics Standards Initiative[3]: Level 1: identified compound confirmed with analysis of authentic standard (spike-in). Level 2: putatively identified compound based on spectral similarity with public/commercial spectral libraries or reference compound in the literature and/or physicochemical properties. Level 3: putatively characterized compound classes. Level 4: unknown compound.

## Supplemental Table 5. Intraclass correlations with 95% confidence intervals for annotated urinary ^1^H NMR metabolites and potassium associated with fruit and vegetable intake, from the first and second 24-hr urine samples, collected on average 3 weeks apart

|  | US (N=2,032) | | UK (N=449) | |
| --- | --- | --- | --- | --- |
|  | r | 95% CI | r | 95% CI |
| 2-hydroxy-2-(4-methyl cyclohex-3-en-1-yl) propoxyglucuronide | 0.39 | 0.35,0.42 | 0.32 | 0.25,0.41 |
| 4-hydroxyhippurate | 0.49 | 0.45,0.52 | 0.39 | 0.31,0.47 |
| Citrate | 0.52 | 0.49,0.55 | 0.65 | 0.60,0.70 |
| Hippurate | 0.55 | 0.52,0.58 | 0.54 | 0.47,0.60 |
| N-methylproline | 0.51 | 0.48,0.55 | 0.59 | 0.53,0.65 |
| Phenylacetylglutamine | 0.60 | 0.57,0.63 | 0.73 | 0.68,0.77 |
| Proline | 0.51 | 0.48,0.55 | 0.50 | 0.43,0.57 |
| Proline betaine | 0.57 | 0.54,0.60 | 0.75 | 0.71,0.76 |
| Scyllo-inositol | 0.58 | 0.55,0.61 | 0.60 | 0.54,0.66 |
| S-methyl-L-cysteine sulfoxide | 0.38 | 0.35,0.42 | 0.70 | 0.65,0.74 |
| S-methyl-L-cysteine sulfoxide metabolite | 0.30 | 0.26,0.34 | 0.50 | 0.43,0.57 |
| Unknown 1 (δ2.47) | 0.62 | 0.60,0.65 | 0.67 | 0.62,0.72 |
| Unknown 2 (δ2.96) | 0.30 | 0.11,0.29 | 0.24 | 0.16,0.34 |
| Unknown 3 (δ2.98) | 0.37 | 0.40,0.55 | 0.47 | 0.40,0.55 |
| Potassium | 0.65 | 0.63,0.68 | 0.59 | 0.52,0.64 |

## Supplemental Table 6. Partial correlations between average fruit and vegetable intake from 4 24-hr dietary recalls with timed 24-hr urinary ^1^H NMR metabolites and potassium in the US, UK and pooled cohort^1^

|  | US (N=2,032) | | UK (N=449) | | US + UK pooled cohort (N=2,481) | | | | | |  |
| --- | --- | --- | --- | --- | --- | --- | --- | --- | --- | --- | --- |
|  | Model 1 | | Model 1 | | Model 1 | | Model 2 | | Model 3 | | |
|  | R | P-value | R | P-value | R | P-value | R | P-value | R | P-value | |
| 2-hydroxy-2-(4-methyl cyclohex-3-en-1-yl) propoxyglucuronide | 0.27 | 3.3x10^-34^ | 0.11 | 1.7x10^-2^ | 0.24 | 1.7x10^-34^ | 0.23 | 4.9x10^-32^ | 0.23 | 1.0x10^-30^ | |
| 4-hydroxyhippurate | 0.16 | 9.1x10^-14^ | 0.14 | 3.2x10^-3^ | 0.16 | 6.9x10^-16^ | 0.17 | 2.3x10^-17^ | 0.16 | 2.2x10^-15^ | |
| Citrate | 0.45 | 3.5x10^-99^ | 0.34 | 2.6x10^-13^ | 0.43 | 8.1x10^-113^ | 0.41 | 2.0x10^-99^ | 0.40 | 2.3x10^-97^ | |
| Hippurate | 0.17 | 3.9x10^-14^ | 0.25 | 5.0x10^-8^ | 0.18 | 2.2x10^-19^ | 0.17 | 1.4x10^-17^ | 0.16 | 2.5x10^-15^ | |
| N-methylproline | 0.35 | 2.3x10^-60^ | 0.34 | 1.7x10^-13^ | 0.35 | 1.4x10^-72^ | 0.36 | 3.3x10^-75^ | 0.36 | 9.9x10^-75^ | |
| Phenylacetylglutamine | -0.11 | 1.7x10^-6^ | -0.12 | 1.0x10^-2^ | -0.11 | 1.1x10^-7^ | -0.12 | 5.6x10^-10^ | -0.12 | 9.3x10^-9^ | |
| Proline | 0.27 | 9.5x10^-35^ | 0.40 | 1.0x10^-18^ | 0.28 | 4.5x10^-46^ | 0.27 | 7.9x10^-42^ | 0.26 | 1.1x10^-39^ | |
| Proline betaine | 0.50 | 2.3x10^-127^ | 0.52 | 1.4x10^-32^ | 0.50 | 1.2x10^-157^ | 0.49 | 1.4x10^-150^ | 0.49 | 2.2x10^-148^ | |
| Scyllo-inositol | 0.36 | 3.8x10^-64^ | 0.35 | 6.3x10^-14^ | 0.36 | 3.4x10^-77^ | 0.33 | 2.7x10^-65^ | 0.33 | 2.9x10^-62^ | |
| S-methyl-L-cysteine sulfoxide | 0.24 | 3.4x10^-27^ | 0.11 | 1.6x10^-2^ | 0.14 | 6.9x10^-13^ | 0.13 | 1.0x10^-10^ | 0.12 | 2.4x10^-9^ | |
| S-methyl-L-cysteine sulfoxide metabolite | 0.27 | 1.5x10^-34^ | 0.19 | 6.0x10^-5^ | 0.26 | 1.3x10^-38^ | 0.24 | 2.0x10^-34^ | 0.23 | 5.9x10^-32^ | |
| Unknown 1 (δ2.47) | -0.24 | 3.6x10^-28^ | -0.23 | 8.0x10^-7^ | -0.24 | 8.7x10^-34^ | -0.23 | 3.8x10^-31^ | -0.22 | 5.0x10^-28^ | |
| Unknown 2 (δ2.96) | 0.32 | 2.0x10^-48^ | 0.27 | 7.3x10^-9^ | 0.28 | 3.1x10^-45^ | 0.27 | 1.4x10^-41^ | 0.26 | 5.2x10^-40^ | |
| Unknown 3 (δ2.98) | 0.29 | 2.4x10^-40^ | 0.21 | 1.2x10^-5^ | 0.27 | 1.2x10^-42^ | 0.26 | 1.3x10^-39^ | 0.26 | 2.2x10^-39^ | |
| Potassium | 0.34 | 1.5x10^-56^ | 0.34 | 7.4x10^-14^ | 0.34 | 6.9x10^-69^ | 0.35 | 7.0x10^-72^ | 0.34 | 5.4x10^-69^ | |

^1^Statistical significance is based at the metabolome-wide significance level (P≤4x10^-6^) for the US and pooled cohort and at the False Discovery Rate (P≤0.05) for the UK cohort. Model 1 was adjusted for age, sex and population sample. Model 2 was adjusted as model 1 plus BMI. Model 3 was adjusted as model 2 plus intake of energy and alcohol, smoking status, years of education, physical activity during leisure time, use of dietary supplements, use of any special diet, history of cardiovascular disease or diabetes mellitus, use of antihypertensive, cardiovascular disease or diabetes medication.

## Supplemental Table 7. Estimated mean differences in systolic and diastolic BP and BMI with a 2SD higher excretion of individual fruit and vegetable-related metabolites and the metabolite score in the US and UK pooled INTERMAP cohort (N=2,481)^1^

|  | Systolic BP | | | Diastolic BP | | | BMI | | |
| --- | --- | --- | --- | --- | --- | --- | --- | --- | --- |
|  | Difference | 95% CI | *P* | Difference | 95% CI | *P* | Difference | 95% CI | *P* |
| 2-hydroxy-2-(4-methyl cyclohex-3-en-1-yl) propoxyglucuronide | | | | | |  |  |  |  |
| Model 1 | -1.35 | -2.40,-0.30 | 0.01 | -0.05 | -0.77,0.66 | 0.89 | -0.69 | -1.11,-0.26 | 1.8x10^-3*^ |
| Model 2 | -1.20 | -2.20,-0.19 | 0.02 | -0.14 | -0.84,0.56 | 0.70 | -0.48 | -0.88,-0.07 | 0.02 |
| Model 3 | -1.09 | -2.09,-0.09 | 0.03 | -0.10 | -0.79,0.60 | 0.78 | -0.40 | -0.80,0.00 | 0.05 |
| Model 4 | -1.04 | -2.05,-0.03 | 0.04 | -0.08 | -0.77,0.62 | 0.83 | -0.32 | -0.72,0.07 | 0.11 |
| Model 5 | -0.83 | -1.80,0.15 | 0.10 | 0.06 | -0.63,0.74 | 0.87 |  |  |  |
| 4-hydroxyhippurate | |  |  |  |  |  |  |  |  |
| Model 1 | -1.96 | -3.01,-0.92 | 2.4x10^-4*^ | -0.55 | -1.27,0.17 | 0.13 | -0.98 | -1.41,-0.54 | 1.1x10^-5*^ |
| Model 2 | -1.56 | -2.56,-0.56 | 2.2x10^-3*^ | -0.35 | -1.04,0.35 | 0.33 | -0.79 | -1.20,-0.39 | 1.4x10^-4*^ |
| Model 3 | -1.34 | -2.33,-0.34 | 0.01 | -0.30 | -1.00,0.39 | 0.39 | -0.60 | -1.00,-0.19 | 3.8x10^-3^ |
| Model 4 | -1.33 | -2.32,-0.33 | 0.01 | -0.29 | -0.99,0.40 | 0.41 | -0.60 | -0.99,-0.20 | 2.9x10^-3*^ |
| Model 5 | -0.95 | -1.92,0.01 | 0.05 | -0.09 | -0.77,0.59 | 0.80 |  |  |  |
| Citrate |  |  |  |  |  |  |  |  |  |
| Model 1 | -4.08 | -5.15,-3.02 | 6.1x10^-14*^ | -1.63 | -2.37,-0.90 | 1.4x10^-5*^ | -2.12 | -2.57,-1.67 | 1.8x10^-13*^ |
| Model 2 | -2.82 | -3.87,-1.77 | 1.4x10^-7*^ | -1.14 | -1.88,-0.41 | 2.2x10^-3^ | -1.60 | -2.04,-1.17 | 3.9x10^-13*^ |
| Model 3 | -2.50 | -3.55,-1.44 | 3.6x10^-6*^ | -1.02 | -1.76,-0.28 | 6.9x10^-3^ | -1.38 | -1.81,-0.95 | 3.9x10^-10*^ |
| Model 4 | -2.46 | -3.52,-1.40 | 5.2x10^-6*^ | -1.00 | -1.74,-0.26 | 8.4x10^-3^ | -1.32 | -1.74,-0.89 | 9.6x10^-10*^ |
| Model 5 | -1.64 | -2.68,-0.61 | 1.9x10^-3*^ | -0.52 | -1.25,0.21 | 0.16 |  |  |  |
| Hippurate | |  |  |  |  |  |  |  |  |
| Model 1 | -4.07 | -5.13,-3.01 | 5.3x10^-14*^ | -1.82 | -2.55,-1.09 | 9.6x10^-7*^ | -2.20 | -2.63,-1.76 | 3.2x10^-15*^ |
| Model 2 | -2.93 | -3.95,-1.91 | 2.0x10^-8*^ | -1.24 | -1.95,-0.53 | 6.6x10^-4*^ | -1.65 | -2.07,-1.24 | 6.2x10^-15*^ |
| Model 3 | -2.55 | -3.57,-1.52 | 1.2x10^-6*^ | -1.00 | -1.71,-0.28 | 6.4x10^-3^ | -1.48 | -1.90,-1.07 | 2.3x10^-12*^ |
| Model 4 | -2.59 | -3.62,-1.56 | 7.8x10^-7*^ | -1.02 | -1.73,-0.30 | 5.5x10^-3^ | -1.54 | -1.95,-1.14 | 6.4x10^-14*^ |
| Model 5 | -1.64 | -2.64,-0.63 | 1.4x10^-3*^ | -0.50 | -1.21,0.21 | 0.17 |  |  |  |
| N-methylproline | |  |  |  |  |  |  |  |  |
| Model 1 | -0.52 | -1.60,0.55 | 0.34 | 0.20 | -0.53,0.94 | 0.59 | -0.77 | -1.22,-0.33 | 7.1x10^-4*^ |
| Model 2 | -0.39 | -1.41,0.63 | 0.45 | 0.23 | -0.48,0.94 | 0.53 | -0.77 | -1.19,-0.35 | 3.2x10^-4*^ |
| Model 3 | -0.39 | -1.41,0.63 | 0.45 | 0.23 | -0.48,0.94 | 0.53 | -0.76 | -1.17,-0.35 | 3.1x10^-4*^ |
| Model 4 | -0.42 | -1.43,0.60 | 0.42 | 0.22 | -0.49,0.93 | 0.54 | -0.83 | -1.23,-0.42 | 5.9x10^-5*^ |
| Model 5 | 0.10 | -0.88,1.09 | 0.84 | 0.50 | -0.20,1.19 | 0.16 |  |  |  |
| Phenylacetylglutamine | |  |  |  |  |  |  |  |  |
| Model 1 | -2.32 | -3.42,-1.23 | 3.0x10^-5*^ | -1.23 | -1.98,-0.48 | 1.2x10^-3*^ | -0.97 | -1.42,-0.53 | 1.9x10^-5*^ |
| Model 2 | -2.20 | -3.25,-1.16 | 3.5x10^-5*^ | -1.15 | -1.88,-0.43 | 1.8x10^-3*^ | -0.93 | -1.47,-0.50 | 1.4x10^-5*^ |
| Model 3 | -2.38 | -3.42,-1.34 | 7.3x10^-6*^ | -1.19 | -1.92,-0.47 | 1.2x10^-3*^ | -1.06 | -1.48,-0.64 | 6.0x10^-7*^ |
| Model 4 | -2.33 | -3.37,-1.29 | 1.2x10^-5*^ | -1.18 | -1.91,-0.46 | 1.4x10^-3*^ | -0.95 | -1.36,-0.55 | 4.4x10^-6*^ |
| Model 5 | -1.70 | -2.71,-0.69 | 1.0x10^-4*^ | -0.83 | -1.54,-0.12 | 2.3x10^-2^ |  |  |  |
| Proline |  |  |  |  |  |  |  |  |  |
| Model 1 | 0.85 | -0.23,1.93 | 0.12 | 0.31 | -0.43,1.05 | 0.41 | 0.38 | -0.07,0.84 | 0.10 |
| Model 2 | 0.67 | -0.38,1.73 | 0.21 | 0.37 | -0.36,1.11 | 0.32 | 0.33 | -0.10,0.77 | 0.14 |
| Model 3 | 0.88 | -0.16,1.93 | 0.10 | 0.45 | -0.28,1.18 | 0.23 | 0.45 | 0.02,0.88 | 0.04 |
| Model 4 | 0.92 | -0.13,1.97 | 0.08 | 0.48 | -0.25,1.22 | 0.20 | 0.47 | 0.05,0.89 | 0.03 |
| Model 5 | 0.62 | -0.39,1.64 | 0.23 | 0.31 | -0.41,1.02 | 0.40 |  |  |  |
| Proline betaine | |  |  |  |  |  |  |  |  |
| Model 1 | -1.52 | -2.57,-0.47 | 4.5x10^-3^ | -0.16 | -0.88,0.55 | 0.65 | -1.01 | -1.44,-0.57 | 6.1x10^-6*^ |
| Model 2 | -0.91 | -1.93,0.11 | 0.08 | 0.00 | -0.71,0.70 | 0.99 | -0.74 | -1.16,-0.33 | 4.6x10^-4*^ |
| Model 3 | -0.73 | -1.74,0.28 | 0.16 | 0.07 | -0.63,0.78 | 0.84 | -0.64 | -1.05,-0.23 | 2.2x10^-3^ |
| Model 4 | -0.64 | -1.66,0.37 | 0.21 | 0.11 | -0.60,0.82 | 0.77 | -0.49 | -0.89,-0.09 | 0.02 |
| Model 5 | -0.30 | -1.28,0.68 | 0.55 | 0.31 | -0.38,1.00 | 0.38 |  |  |  |
| Scyllo-inositol | |  |  |  |  |  |  |  |  |
| Model 1 | -0.96 | -2.19,0.27 | 0.13 | -0.35 | -1.19,0.49 | 0.42 | -0.55 | -1.07,-0.04 | 0.04 |
| Model 2 | -0.61 | -1.80,0.59 | 0.32 | -0.15 | -0.98,0.69 | 0.73 | -0.32 | -0.81,0.17 | 0.20 |
| Model 3 | -0.42 | -1.62,0.77 | 0.49 | -0.08 | -0.91,0.75 | 0.84 | -0.19 | -0.68,0.29 | 0.44 |
| Model 4 | -0.32 | -1.52,0.87 | 0.59 | -0.04 | -0.88,0.79 | 0.92 | 0.00 | -0.47,0.47 | 1.00 |
| Model 5 | -0.30 | -1.45,0.86 | 0.61 | -0.03 | -0.84,0.78 | 0.94 |  |  |  |
| S-methyl-L-cysteine sulfoxide | | |  |  |  |  |  |  |  |
| Model 1 | -0.45 | -1.50,0.61 | 0.41 | -0.15 | -0.86,0.57 | 0.69 | -0.19 | -0.57,0.19 | 0.32 |
| Model 2 | -0.20 | -1.21,0.82 | 0.70 | -0.06 | -0.75,0.63 | 0.86 | -0.19 | -0.55,0.17 | 0.31 |
| Model 3 | -0.03 | -1.04,0.99 | 0.96 | -0.08 | -0.77,0.61 | 0.82 | -0.08 | -0.44,0.28 | 0.67 |
| Model 4 | -0.04 | -1.05,0.98 | 0.94 | -0.06 | -0.75,0.63 | 0.86 | -0.13 | -0.49,0.22 | 0.46 |
| Model 5 | 0.05 | -0.92,1.02 | 0.92 | -0.03 | -0.71,0.65 | 0.93 |  |  |  |
| S-methyl-L-cysteine sulfoxide metabolite | | |  |  |  |  |  |  |  |
| Model 1 | -3.35 | -4.39,-2.30 | 3.8x10^-10*^ | -1.70 | -2.42,-0.98 | 3.7x10^-6*^ | -1.86 | -2.31,-1.41 | 2.2x10^-16*^ |
| Model 2 | -2.29 | -3.30,-1.27 | 9.9x10^-6*^ | -1.26 | -1.97,-0.56 | 4.5x10^-4*^ | -1.40 | -1.82,-0.98 | 6.9x10^-11*^ |
| Model 3 | -2.06 | -3.07,-1.05 | 6.3x10^-5*^ | -1.16 | -1.86,-0.45 | 1.3x10^-3*^ | -1.28 | -1.70,-0.87 | 1.5x10^-9*^ |
| Model 4 | -2.01 | -3.02,-1.00 | 9.9x10^-5*^ | -1.13 | -1.84,-0.42 | 1.8x10^-3*^ | -1.20 | -1.61,-0.79 | 6.9x10^-9*^ |
| Model 5 | -1.28 | -2.26,-0.29 | 1.1x10^-2^ | -0.71 | -1.41,-0.01 | 4.6x10^-2^ |  |  |  |
| Unknown1 (δ2.47) | |  |  |  |  |  |  |  |  |
| Model 1 | -0.05 | -1.19,1.09 | 0.93 | 0.39 | -0.39,1.17 | 0.33 | 0.43 | -0.04,0.90 | 0.07 |
| Model 2 | 0.91 | -0.22,2.04 | 0.11 | 0.71 | -0.07,1.49 | 0.07 | 0.73 | 0.27,1.18 | 1.7x10^-3*^ |
| Model 3 | 0.60 | -0.52,1.72 | 0.29 | 0.52 | -0.26,1.30 | 0.19 | 0.65 | 0.20,1.10 | 4.6x10^-3*^ |
| Model 4 | 0.57 | -0.56,1.69 | 0.32 | 0.50 | -0.28,1.29 | 0.21 | 0.60 | 0.16,1.04 | 8.1x10^-3*^ |
| Model 5 | 0.18 | -0.91,1.27 | 0.75 | 0.30 | -0.47,1.07 | 0.44 |  |  |  |
| Unknown2 (δ2.96) | |  |  |  |  |  |  |  |  |
| Model 1 | -0.99 | -2.11,0.13 | 0.08 | -0.06 | -0.82,0.70 | 0.87 | -0.86 | -1.31,-0.41 | 1.7x10^-4*^ |
| Model 2 | -0.72 | -1.79,0.35 | 0.19 | -0.08 | -0.82,0.66 | 0.83 | -0.77 | -1.19,-0.34 | 3.8x10^-4*^ |
| Model 3 | -0.73 | -1.79,0.33 | 0.18 | -0.10 | -0.83,0.64 | 0.80 | -0.75 | -1.17,-0.33 | 4.2x10^-4*^ |
| Model 4 | -0.73 | -1.79,0.34 | 0.18 | -0.10 | -0.84,0.64 | 0.79 | -0.77 | -1.18,-0.36 | 2.4x10^-4*^ |
| Model 5 | -0.22 | -1.25,0.81 | 0.67 | 0.16 | -0.56,0.88 | 0.67 |  |  |  |
| Unknown3 (δ2.98) | |  |  |  |  |  |  |  |  |
| Model 1 | -0.07 | -1.28,1.13 | 0.91 | 0.52 | -0.29,1.34 | 0.21 | -0.01 | -0.46,0.45 | 0.98 |
| Model 2 | -0.08 | -1.24,1.07 | 0.89 | 0.35 | -0.44,1.14 | 0.39 | -0.09 | -0.52,0.35 | 0.69 |
| Model 3 | -0.10 | -1.25,1.06 | 0.87 | 0.25 | -0.54,1.04 | 0.53 | 0.01 | -0.42,0.44 | 0.96 |
| Model 4 | -0.11 | -1.27,1.05 | 0.86 | 0.26 | -0.54,1.05 | 0.83 | -0.05 | -0.48,0.37 | 0.81 |
| Model 5 | -0.10 | -1.21,1.01 | 0.86 | 0.24 | -0.53,1.02 | 0.54 |  |  |  |
| Potassium |  |  |  |  |  |  |  |  |  |
| Model 1 | -1.22 | -2.37,-0.07 | 0.04 | -0.88 | -1.67,-0.09 | 0.03 | 1.00 | 0.53,1.47 | 3.3x10^-5*^ |
| Model 2 | -1.31 | -2.45,-0.17 | 0.02 | -0.89 | -1.68,-0.10 | 0.03 | 1.20 | 0.75,1.66 | 2.5x10^-7*^ |
| Model 3 | -0.90 | -2.09,0.29 | 0.14 | -0.40 | -1.23,0.42 | 0.34 | 1.30 | 0.82,1.77 | 7.4x10^-8*^ |
| Model 4 | -1.52 | -2.79,-0.24 | 0.02 | -0.60 | -1.49,0.28 | 0.18 | 0.51 | 0.01,1.00 | 0.04 |
| Model 5 | -1.80 | -2.96,-0.64 | 2.3x10^-3*^ | -0.90 | -1.72,-0.09 | 0.03 |  |  |  |
| Metabolite score (2SD = 0.77) | | | | |  |  |  |  |  |
| Model 1 | -3.70 | -4.78,-2.62 | 1.7x10^-11*^ | -1.23 | -1.97,-0.48 | 1.2x10^-3*^ | -1.75 | -2.19,-1.30 | 1.4x10^-14*^ |
| Model 2 | -2.72 | -3.78,-1.66 | 4.8x10^-7*^ | -0.84 | -1.58,-0.11 | 0.02 | -1.33 | -1.76,-0.90 | 1.5x10^-9*^ |
| Model 3 | -2.38 | -3.44,-1.32 | 1.0x10^-5*^ | -0.67 | -1.41,0.06 | 0.07 | -1.14 | -1.56,-0.71 | 1.8x10^-7*^ |
| Model 4 | -2.41 | -3.47,-1.34 | 9.3x10^-6*^ | -0.67 | -1.41,0.07 | 0.08 | -1.21 | -1.62,-0.78 | 1.7x10^-8*^ |
| Model 5 | -1.65 | -2.68,-0.62 | 1.7x10^-3*^ | -0.27 | -1.00,0.46 | 0.47 |  |  |  |

^1^ Urinary metabolite excretions were mean of two timed 24-hr urine collections. Differences in BP and BMI and corresponding 95% confidence intervals were obtained from multivariable linear regression analyses by pooling cross-country regression coefficients weighted by sample size. Model 1 was adjusted for age, sex and population sample. Model 2 was adjusted as model 1 plus intake of energy (kcal/day) and alcohol (g/day), smoking status (never, former, current), years of education (years completed), physical activity during leisure time (a lot, moderate, little or none), use of dietary supplements (yes/no), use of any special diet (yes/no), history of cardiovascular disease or diabetes mellitus (yes/no), family history of cardiovascular disease (yes/no), and use of antihypertensive, cardiovascular disease or diabetes medication (yes/no). Model 3 was adjusted as model 2 plus intake (g/1000 kcal) of low-fat dairy products, fiber-rich cereals and grains, red and processed meats, and fish and shellfish. Model 4 was adjusted as model 3 plus urinary sodium excretion (mmol/24-hr). Model 5 was adjusted as model 3 plus BMI (for systolic and diastolic BP only). Cross-country heterogeneity of regression coefficients was assessed by chi-square test: no significant heterogeneity across countries was detected. A Bonferroni correction was applied resulting in a significance level of *P*≤3.0x10^-3^; ^*^ indicates significant associations.

## Supplemental Table 8. Estimated mean differences in systolic and diastolic BP and BMI with a 2SD increase in metabolite score with additional adjustment for each individual metabolite or urinary potassium in the US and UK pooled INTERMAP cohort (N=2,481)^1^

|  | Systolic BP | | | Diastolic BP | | | BMI | | |
| --- | --- | --- | --- | --- | --- | --- | --- | --- | --- |
|  | Difference | 95% CI | *P* | Difference | 95% CI | *P* | Difference | 95% CI | *P* |
| **Metabolite score** | -2.38 | -3.44,-1.32 | 1.0x10^-5*^ | -0.67 | -1.41,0.06 | 0.07 | -1.14 | -1.56,-0.71 | 1.8x10^-7*^ |
| + 2-hydroxy-2-(4-methyl cyclohex-3-en-1-yl) propoxyglucuronide | -2.43 | -3.67,-1.19 | 1.2x10^-4*^ | -0.87 | -1.73,-0.04 | 0.05 | -1.14 | -1.63,-0.64 | 7.0x10^-6*^ |
| + 4-hydroxyhippurate | -2.15 | -3.27,-1.02 | 2.0x10^-4*^ | -0.65 | -1.44,0.13 | 0.10 | -1.00 | -1.45,-0.54 | 2.0x10^-5*^ |
| + Citrate | -1.23 | -2.75,0.30 | 0.11 | 0.01 | -1.07,1.06 | 0.99 | -0.23 | -0.84,0.38 | 0.45 |
| + Hippurate | -1.82 | -2.91,-0.73 | 1.1x10^-3*^ | -0.45 | -1.22,0.31 | 0.24 | -0.74 | -1.18,-0.31 | 8.8x10^-4*^ |
| + N-methylproline | -4.05 | -5.06,-2.59 | 5.4x10^-8*^ | -1.63 | -2.65,-0.62 | 1.6x10^-3*^ | -1.07 | -1.66,-0.48 | 3.6x10^-4*^ |
| + Phenylacetylglutamine | -2.21 | -3.27,-1.16 | 4.0x10^-5*^ | -0.60 | -1.34,0.14 | 0.11 | -1.02 | -1.45,-0.59 | 2.8x10^-6*^ |
| + Proline | -3.30 | -4.45,-2.15 | 1.9x10^-8*^ | -1.05 | -1.85,-0.25 | 1.0x10^-2^ | -1.54 | -2.00,-1.07 | 8.4x10^-11*^ |
| + Proline betaine | -4.77 | -6.51,-3.03 | 7.6x10^-8*^ | -2.00 | -3.22,-0.79 | 1.2x10^-3*^ | -1.56 | -2.26,-0.86 | 1.17x10^-5*^ |
| + Scyllo-inositol | -3.00 | -4.24,-1.77 | 1.8x10^-6*^ | -0.88 | -1.74,-0.02 | 0.04 | -1.39 | -1.89,-0.90 | 4.2x10^-8*^ |
| + S-methyl-L-cysteine sulfoxide | -2.48 | -3.57,-1.38 | 9.3x10^-6*^ | -0.72 | -1.48,0.05 | 0.07 | -1.17 | -1.61,-0.72 | 2.7x10^-7*^ |
| + S-methyl-L-cysteine sulfoxide metabolite | -1.78 | -2.98,-0.58 | 3.5x10^-3^ | -0.19 | -1.02,0.64 | 0.65 | -0.61 | -1.09,-0.13 | 1.3x10^-2^ |
| + Unknown 1 (δ2.47) | -2.23 | -3.40,-1.27 | 1.7x10^-5*^ | -0.65 | -1.39,0.10 | 0.09 | -1.03 | -1.46,-0.60 | 2.8x10^-6*^ |
| + Unknown 2 (δ2.96) | -2.66 | -3.89,-1.43 | 2.2x10^-5*^ | -0.80 | -1.65,0.06 | 0.07 | -0.97 | -0.46,-0.47 | 1.4x10^-4*^ |
| + Unknown 3 (δ2.98) | -3.14 | -4.42,-1.85 | 1.9x10^-6*^ | -1.02 | -1.92,-0.12 | 0.03 | -1.69 | -2.21,-1.17 | 2.5x10^-10*^ |
| + Potassium | -2.42 | -3.55,-1.28 | 3.1 x10^-5*^ | -0.63 | -1.43,0.16 | 0.12 | -1.80 | -2.25,-1.34 | 9.3 x10^-15*^ |

**^1^** Urinary metabolite excretions were mean of two 24-hr urine collections. Differences in BP and BMI and corresponding 95% confidence intervals were obtained from multivariable linear regression analyses by pooling cross-country regression coefficients weighted by sample size. Analyses were fully adjusted (model 3) including age, sex, population sample, intake of energy (kcal/day) and alcohol (g/day), smoking status (never, former, current), years of education (years completed), physical activity during leisure time (a lot, moderate, little or none), use of dietary supplements (yes/no), use of any special diet (yes/no), history of cardiovascular disease or diabetes mellitus (yes/no), family history of cardiovascular disease (yes/no), and use of antihypertensive, cardiovascular disease or diabetes medication (yes/no), intake (g/1000 kcal) of low-fat dairy products, fibre-rich cereals and grains, red and processed meats, and fish and shellfish, and additionally for each individual metabolite or urinary potassium. Cross-country heterogeneity of regression coefficients was assessed by chi-square test: no significant heterogeneity across countries was detected. A Bonferroni correction was applied resulting in a significance level of *P*≤3.0x10^-3^; ^*^ indicates significant associations.

## Supplemental Table 9. Estimated mean differences in systolic and diastolic BP and BMI with a 2SD increase in average metabolite score from two 24-hr urine collections in subcohorts^1^

|  | | Systolic BP | | | | | | Diastolic BP | | | BMI | | |
| --- | --- | --- | --- | --- | --- | --- | --- | --- | --- | --- | --- | --- | --- |
|  | | Difference | 95% CI | | *P* | | | Difference | 95% CI | *P* | Difference | 95% CI | *P* |
| Excluding participants with self-reported diagnosis of hypertension or  on antihypertensive treatment (N=1,700) | | | | | | | | |  |  |  |  |  |
| Model 3 | -2.55 | | | -3.65,-1.45 | | 5.6x10^-6*^ | -0.86 | | -1.67,-0.05 | 0.04 | -1.12 | -1.61,-0.64 | 5.8x10^-6*^ |
| Model 3 + BMI | -1.74 | | | -2.80,-0.69 | | 1.1x10^-3*^ | -0.33 | | -1.12,0.45 | 0.40 |  |  |  |
| Nonhypertensive participants (N=1,627) | | | | | |  |  | |  |  |  |  |  |
| Model 3 | -2.40 | | | -3.38,-1.42 | | 1.7x10^-6*^ | -0.86 | | -1.61,-0.10 | 0.03 | -1.11 | -1.59,-0.64 | 4.95x10^-6*^ |
| Model 3 + BMI | -1.74 | | | -2.69,-0.79 | | 3.4x10^-4*^ | -0.40 | | -1.13,0.34 | 0.29 |  |  |  |
| Further exclusion of participants with self-reported diagnosis of cardiovascular diseases or diabetes mellitus (N=1,477) | | | | | | | | |  |  |  |  |  |
| Model 3 | -2.36 | | | -3.39,-1.34 | | 6.2x10^-6*^ | -0.90 | | -1.67,-0.13 | 0.02 | -1.22 | -1.72,-0.73 | 1.5x10^-6*^ |
| Model 3 + BMI | -1.64 | | | -2.63,-0.65 | | 1.2x10^-3*^ | -0.38 | | -1.13,0.37 | 0.32 |  |  |  |

^1^ Differences in BP and BMI and corresponding 95% confidence intervals were obtained from multivariable linear regression analyses by pooling cross-country regression coefficients weighted by sample size. Model 3 was adjusted for age, sex, population sample, intake of energy (kcal/day) and alcohol (g/day), smoking status (never, former, current), years of education (years completed), physical activity during leisure time (a lot, moderate, little or none), use of dietary supplements (yes/no), use of any special diet (yes/no), history of cardiovascular disease or diabetes mellitus (yes/no), family history of cardiovascular disease (yes/no), use of antihypertensive, cardiovascular disease or diabetes medication (yes/no), intake (g/1000 kcal) of low-fat dairy products, fiber-rich cereals and grains, red and processed meats, and fish and shellfish. Cross-country heterogeneity of regression coefficients was assessed by chi-square test: no significant heterogeneity across countries was detected. A Bonferroni correction was applied with a significance level of *P*≤3.0x10^-3^; ^*^ indicates significant associations.

## References

1. E. Holmes, R.L. Loo, J. Stamler, M. Bictash, I.K. Yap, Q. Chan, et al., Human metabolic phenotype diversity and its association with diet and blood pressure, Nature. 453 (7193) (2008) 396-400

2. J. Textor, B. van der Zander, M.S. Gilthorpe, M. Liskiewicz, G.T. Ellison, Robust causal inference using directed acyclic graphs: the R package 'dagitty', Int J Epidemiol. 45 (6) (2016) 1887-1894

3. L.W. Sumner, A. Amberg, D. Barrett, M.H. Beale, R. Beger, C.A. Daykin, et al., Proposed minimum reporting standards for chemical analysis Chemical Analysis Working Group (CAWG) Metabolomics Standards Initiative (MSI), Metabolomics : Official journal of the Metabolomic Society. 3 (3) (2007) 211-221
